# Soluble CD83 Triggers Resolution of Arthritis and Sustained Inflammation Control in IDO Dependent Manner

**DOI:** 10.3389/fimmu.2019.00633

**Published:** 2019-04-02

**Authors:** Dmytro Royzman, Darja Andreev, Lena Stich, Manfred Rauh, Tobias Bäuerle, Stephan Ellmann, Louis Boon, Markus Kindermann, Katrin Peckert, Aline Bozec, Georg Schett, Alexander Steinkasserer, Elisabeth Zinser

**Affiliations:** ^1^Department of Immune Modulation, Universitätsklinikum Erlangen, Erlangen, Germany; ^2^Department of Internal Medicine 3, Universitätsklinikum Erlangen, Erlangen, Germany; ^3^Department of Pediatrics and Adolescent Medicine, Universitätsklinikum Erlangen, Erlangen, Germany; ^4^Institute of Radiology, Universitätsklinikum Erlangen, Erlangen, Germany; ^5^Bioceros, Utrecht, Netherlands; ^6^Department of Internal Medicine 1, Universitätsklinikum Erlangen, Erlangen, Germany

**Keywords:** soluble CD83, arthritis, IDO, osteoclasts, Tregs

## Abstract

Interference with autoimmune-mediated cytokine production is a key yet poorly developed approach to treat autoimmune and inflammatory diseases such as rheumatoid arthritis. Herein, we show that soluble CD83 (sCD83) enhances the resolution of autoimmune antigen-induced arthritis (AIA) by strongly reducing the expression levels of cytokines such as IL-17A, IFNγ, IL-6, and TNFα within the joints. Noteworthy, also the expression of RANKL, osteoclast differentiation, and joint destruction was significantly inhibited by sCD83. In addition, osteoclasts which were cultured in the presence of synovial T cells, derived from sCD83 treated AIA mice, showed a strongly reduced number of multinuclear large osteoclasts compared to mock controls. Enhanced resolution of arthritis by sCD83 was mechanistically based on IDO, since inhibition of IDO by 1-methyltryptophan completely abrogated sCD83 effects on AIA. Blocking experiments, using anti-TGF-β antibodies further revealed that also TGF-β is mechanistically involved in the sCD83 induced reduction of bone destruction and cartilage damage as well as enhanced resolution of inflammation. Resolution of arthritis was associated with increased numbers of regulatory T cells, which are induced in a sCD83-IDO-TGF-β dependent manner. Taken together, sCD83 represents an interesting approach for downregulating cytokine production, inducing regulatory T cells and inducing resolution of autoimmune arthritis.

## Introduction

Rheumatoid arthritis (RA) is an autoimmune inflammatory disease affecting up to 1% of the population (1). Both epigenetic and environmental factors are considered to promote the disease leading to loss of tolerance to self-antigens (2). RA manifests through local symptoms, such as swelling and pain (3) as well as systemic complications like myocardial infarction (4), atherosclerosis (5), lymphoma (6), and functional disability. Current treatment of RA aims to block pro-inflammatory effector cytokines such as TNFα and IL6, which are produced in the synovium and trigger arthritis (7, 8). However, only a fraction of RA patients responds to these therapies and, even if responding, these treatments have to be given life-long to prevent recurrence of the disease (9). Better approaches to modulate the link between autoimmunity and cytokine productions are therefore needed to allow long-term remission or even cure of RA.

The soluble form of the CD83 molecule (sCD83), which is highly expressed by mature dendritic cells (DC) but also by activated B and T cells and especially Tregs, comprises very interesting immunomodulatory properties (10–13). We and others reported that sCD83 interferes with the maturation process of DCs, thereby limiting immune responses and inducing tolerogenic mechanisms (10, 12–14). DC activation by autoantigens and persistent stimulation of T cells is considered one of the main reasons for impaired resolution of inflammation in RA patients. Thus, by controlling DC maturation/activation, one could regulate immune homeostasis and the balance between tolerance and autoimmunity (15). Furthermore, manipulation of DCs, and their maturation/activation status, i.e., via the induction of immature, tolerogenic DC, and inhibition of mature DC, having activating properties, represents an interesting approach to interfere with the outcome of human inflammatory and autoimmune disorders such as RA.

Furthermore, Tregs play a crucial role during resolution of inflammation and also protect from bone destruction in arthritis (16). Noteworthy, since sCD83 leads to the induction/expansion of Tregs (10, 11) one may not only block long-lasting proinflammatory autoimmune responses, but also induce intrinsic mechanisms leading to the resolution of inflammatory processes. Since Tregs suppress osteoclast differentiation and reduce bone destruction (16), sCD83 may additionally affect osteoclastogenesis and joint destruction by induced Treg cells.

Interestingly, increased levels of sCD83 have been observed in the synovial fluids of rheumatoid arthritis patients (17), indicating a biological role of sCD83 in rheumatoid diseases. In order to investigate whether sCD83 is indeed involved in the modulation of the inflammatory response in RA we studied the effects of sCD83 using models of immune mediated arthritis (18).

## Materials and Methods

### Mice

Female C57BL/6 mice (6–8 weeks old) were purchased from Charles River Laboratories (Sulzfeld) and maintained under pathogen free conditions according to the institutional and national guidelines for the care and use of laboratory animals. All studies were approved by the animal ethical committee of the government of Unterfranken, Würzburg.

### Induction of AIA

Mice were pre-immunized at day −21 and−14 by s.c. injection of 100 μl complete Freund's adjuvant (CFA) emulsion (Sigma-Aldrich) enriched with 10 μg/ml *Mycobacterium tuberculosis* strain H37RA (Difco) and methylated bovine serum albumin (mBSA) (Sigma-Aldrich) in a final concentration of 1 mg/ml. Along with the immunization, 200 ng Bordetella pertussis toxin (Quadratech) was administered i.p. in 100 μl phosphate-buffered saline (PBS) (Lonza). The effector phase was induced on day 0 by the intra-articular (i.a.) injection of 100 μg mBSA into the right knee of anesthetized mice. The left knee was injected with PBS as a control. Knee joint swelling was assessed from the time of induction (day 0) up to day 10 using a JD 50 TOP caliper (Käfer Messuhrenfabrik). In specific experiments a flare up reaction was induced by a second i.a. mBSA injection on day 7, analogous to the first i.a. injection, and the knee swelling was assessed until day 17. The maximum medial-to-lateral diameter was defined at the widest point of each knee joint. Knee joint swelling was calculated as the absolute difference to the knee joint diameter determined at baseline before arthritis induction and expressed as percentage of knee joint swelling. The mice were euthanized by cervical dislocation at day 10 or 17 in case of a flare up reaction. Blood samples were collected on day −19,−14,−3, 3, and 10, centrifuged in microtainer blood collection tubes (BD) and the sera stored at −80°C for further use.

### Serum Transfer Arthritis (STA)

Arthritis was induced by i.p. injection of 200 μl pooled sera from K/BxN mice kindly provided by Wolfgang Baum (Department of Internal Medicine 3, University Hospital, Erlangen, Germany) at day 0 into C57BL/6 mice. Serum was obtained from 8-week-old K/BxN mice, as reported previously (19). On day 31, mice were injected again with 200 μl sera from K/BxN mice. sCD83 treatment during STA was performed by daily i.p. injections (100 μg in 100 μl PBS), starting at day −1 prior serum transfer until day 13. The same amount of PBS was used as control. Joint swelling was examined in all four paws, and a clinical score of 0–3 was assigned (0 = no swelling, 1 = mild, 2 = moderate and 3 = severe swelling of the toes and ankle), as described previously (20). Mice were sacrificed 16 days after the second serum transfer. The left hind paw of each mouse was used for serial paraffin sections.

### *In vitro* Osteoclastogenesis

Total bone marrow cells were isolated from WT BL/6 (7 weeks) mice by flushing femur and tibia. Afterwards, cells were plated overnight in OC medium (αMEM + GlutaMAX (Gibco) + 10%FCS/1%PS) supplemented with 5 ng/ml M-CSF (Peprotech). Non-adherent bone marrow derived monocytes (BMMs) were collected, washed and further cultured in OC medium supplemented with 20 ng/ml M-CSF and 10 ng/ml RANKL (Peprotech) in 96-well- (TRAP) and 48-well plates (RNA) at a density of 1 × 10^6^ cells/ml. Additionally, a control condition with 20 ng/ml M-CSF only was included. Medium was changed every second day. From day 1, cells were incubated daily with 10 or 25 μg/ml sCD83. At day 5 fully differentiated osteoclasts were washed with PBS and fixed with fixation buffer (25 ml citrate buffer + 65 ml acetone + 8 ml 37%PFA). Osteoclast differentiation was evaluated by TRAP staining using the leukocyte acid phosphatase kit 386A (Sigma-Aldrich) according to the manufacturer's instructions. For RNA analyses, cells were harvested in peqGOLD TRIfast (peqlab).

### Immunofluorescence

BMMs were cultured on 12 mm circle cover slips (Thermo Fisher Scientific) in 24-well plates with the above described cell density and stimulation protocol. At day 4 the cells were fixed and stained with Acti-stain 670 phalloidin (Cytoskeleton, Inc.), according to the manufacturer's instructions, to visualize the F-actin ring formation. After washing with PBS, cover slips were mounted with Fluoroshield^TM^ with DAPI (Sigma), and transferred upside down on object slides.

### Resorption Assay

BMMs were cultured on 24- well calcium phosphate-coated plates (Corning) in OC medium with 20 ng/ml M-CSF and 20 ng/ml RANKL. BMMs were stimulated as aforementioned. After 4 days of stimulation, osteoclasts were lysed with dH_2_O and plates were incubated with 5% sodium hypochlorite for 5 min. Plates were washed twice with dH_2_O and dried at room temperature (RT).

### Co-culture With Synovial CD4^+^ Cell

Synovial cells were isolated as described below and CD4^+^ MACS separation was performed using the CD4^+^ T cell isolation kit (mouse) according to manufacturer's protocol (Miltenyi Biotec). BMMs (1x10^6^ cells/ml) were cultured with MACS-separated CD4^+^ cells from the synovium derived from mock or sCD83 treated arthritic mice, at a ratio of 1:10 (1 × 10^5^ CD4^+^ cells/ml) or 1:50 (2 × 10^4^ CD4^+^ cells/ml) in OC medium in the presence of 20 ng/ml M-CSF and 10 ng/ml RANKL. Medium was changed after 2 days. At day 4 osteoclasts were fixed and TRAP stained.

Osteoclast cultures were analyzed using the BZ-X710-All-in-One Fluorescence Microscope (Keyence) and the quantification of the osteoclast number and the percentage of resorbed area was performed using ImageJ.

### Viability Test of Preosteoclasts

BMMs (1 × 10^6^ cells/ml) were cultured in 48-well plates in OC medium supplemented with M-CSF (20 ng/ml) and RANKL (10 ng/ml) or M-CSF (20 ng/ml) alone. At day 1 of culture, BMMs were incubated with 10 or 75 μg/ml sCD83 to exclude possible toxic effects of the protein. After 24 h cells were detached from the plate by incubation with Accutase (Thermo Fisher Scientific) at 37°C for 10 min. Cells were then washed with PBS and incubated with anti-CD16/CD32 blocking antibody (BioLegend) for 10 min at RT, followed by staining with an antibody cocktail for 30 min at 4°C in the dark. The following antibodies were used to detect viability of preosteoclasts: APC-eFluor780-labeled anti-CD45 (eBioscience), APC-labeled anti-F4/80 (BioLegend), PE-labeled anti-OSCAR (Beckman coulter), DAPI (Roche), and added shortly before measurement. Data were acquired on the Gallios flow cytometer (Beckman Counter) and analyzed with the Kaluza software 1.5.

### Treatment Conditions

**Figure 1:**
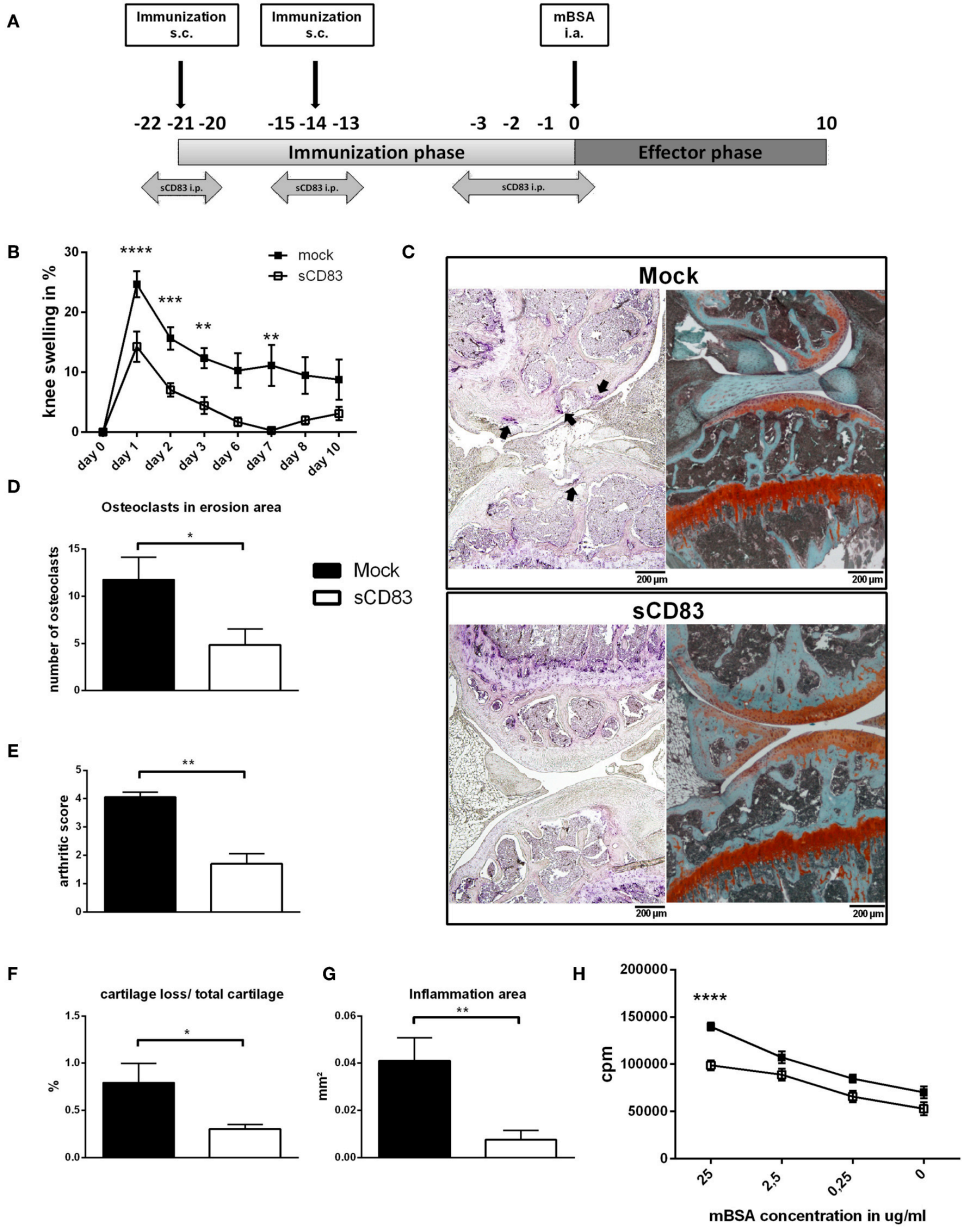
sCD83 ameliorates disease severity in the antigen-induced arthritis model. **(A)** Treatment scheme of sCD83 in the antigen-induced arthritis (AIA) model. **(B)** Percent increase of knee swelling (normalized to baseline) after the local i.a. injection of mBSA and sCD83 treatment (*n* = 15) or vehicle (*n* = 15). Data were pooled from independent experiments. **(C)** Representative 4 μm TRAP (left) and Safranin O (right) stained sections of sCD83 or mock treated mice at day 10. **(D)** Number of osteoclasts located on eroded surfaces (sCD83 *n* = 7, mock *n* = 4). **(E)** Histologic arthritic score based on cartilage degradation, bone resorption and inflammation obtained from Safranin O stained knee joint sections (0 = normal and 5 =severe; sCD83 *n* = 5, mock *n* = 5). **(F,G)** Quantification of the cartilage degradation and inflammation using histomorphometry (sCD83 *n* = 5, mock *n* = 5). **(H)** Antigen specific T cell proliferation of LN cells upon mBSA re-stimulation (sCD83 *n* = 7, mock *n* = 6). Data are shown as mean ± SEM. **(B,H)** Two way ANOVA and (D-G) Mann-Whitney test. Asterisks mark statistically significant difference (^*^p < 0.05, ^**^p < 0.01, ^***^p < 0.001, and ^****^p < 0.0001). The absence of asterisks indicates that there is no statistical significance.

Mice were treated using i.p. injections of sCD83 (100 μg/injection) or the corresponding volume of 100 μl PBS as mock control. The application was performed on day −22,−21,−20,−15,−14,−13,−3,−2,−1, and 0 as illustrated in Figure 1A.

To block the TGF-β signaling pathway, mice were treated from day −21 until day −12 and from day −1 until day 7 with anti α-TGF-β-antibody (150 μg/injection).

To block the activity of indoleamine-2, 3-dioxygenase (IDO) *in vivo*, the inhibitor 1-methyl-DL-tryptophan (1-MT) was applied as polymer pellets impregnated with 1-Methyl-DL-Tryptophan (1-MT) or placebo. Pellets (Innovative Research of America) were inserted surgically at day −22 as previously described (10).

### Micro-CT Analysis

Hind limbs were isolated, fixed overnight in 10% formalin solution at 4°C (Sigma-Aldrich) and stored in 70% ethanol until μCT measurements. Imaging was performed using a dedicated preclinical scanner (Inveon, Siemens Healthineers) at a tube voltage of 80 kV and a tube current of 500 μA. Images were acquired with an isotropic resolution of 50.67 μm for measuring mean density values within the femoral epiphyses, and with 8.98 μm for high- resolution 3D surface reconstructions. For image analyses, 3D multiplanar reconstructions were generated using the freeware DICOM viewer Osirix (21), with a total of 6 regions of interest (ROI) placed in the femoral epiphysis of each animal (2 in paraaxial, 2 in paracoronal, and 2 in parasagittal orientation). Target size of each ROI was 0.5 mm^2^. The mean density measures [Hounsfield units] of the six ROI were assessed and averaged for each animal. For 3D surface reconstructions, the Volume Rendering Technique included in the syngo.via package (Version V20A, Siemens Healthineers,) was used.

### mBSA Specific IgG1 ELISA

A 96-well flat-bottom half area plate (Costar) was coated with 10 μg/ml mBSA in coating buffer (15 mM Na2CO3 and 35 mM NaHCO3) for 1 h and blocked with 2% FCS (PAA Laboratories GmbH) in PBS for 2 h. Serum from AIA mice (day 10) was diluted 1:150 with blocking buffer and incubated for 2 h. HRP conjugated goat anti-Mouse IgG1-antibody (Bethyl) was diluted 1:10.000 in blocking buffer and loaded for further 1 h. The horseradish peroxidase activity was induced by TMB-substrate (Merck), stopped with 6% orthophosphoric acid and the signal was assessed using a Wallac 1420 Victor2 Microplate Reader (Perkin Elmer) at 450 nm. All incubation steps were performed at RT.

### Isolation of Synovial Cells

Synovial cells were isolated using the Multi Tissue Dissociation Kit 1 (Miltenyi Biotec) according to manufacturer's protocol. In brief, the synovial tissue was isolated and placed into gentleMACS C Tubes (Miltenyi Biotec) containing 2.35 ml RPMI (Lonza), 100 μl enzyme D, 50 μl enzyme R und 12.5 μl enzyme A. Program 7C_Multi_A was ran on the gentleMACS Octo Dissociator (Miltenyi Biotec). After homogenization the cell suspension was applied on MACS SmartStrainer (Miltenyi Biotec) and centrifuged for 10 min at 300 g. Cells were then resuspended in 1 ml R10 culture medium (RPMI 1640 (Lonza) supplemented with 100 U/ml penicillin, 100 μg/ml streptomycin, 2 mM L-glutamine (L-Glutamine–Penicillin–Streptomycin solution, Sigma-Aldrich), 50 μM 2-mercaptoethanol (Sigma-Aldrich), and 10% heat-inactivated FCS (Merck) and used for restimulation assays and flow cytometric analyses.

### Antigen Specific Re-stimulation

On day 10, inguinal lymph node (LN) cells and synovial cells were harvested. After preparation of single cell suspensions, cells were resuspended in R10 Medium. LN cells and synovial cells (each 4 × 10^5^) were cultured in the presence of indicated amounts of mBSA (25, 2.5, and 0.25 μg/ml) in 96-well plates for 72 h, followed by the incorporation with 0.2 Ci/mmol Thymidine [3H] (PerkinElmer). The plates were harvested using the ICH-110 harvester (Inotech) and the antigen specific cell proliferation was determined using a 1450-microplate counter (Wallac).

### Intracellular Cytokine Expression of LN and Synovial Cells

Naïve synovial cells were used to analyze their Foxp3 expression profile, while the expression of IL-17A and IFNγ was assessed after *in vitro* stimulation of 8 × 10^6^ cells with 20 ng/ml PMA (Sigma-Aldrich) and 1 μg/ml Ionomycin (Sigma-Aldrich) in a 6-well flat-bottom plate (Falcon) for 6 h. To prevent cytokine GolgiPlug (BD) (1 μl/ml) and GolgiStop (0,6 μl/ml) (BD) were added after the first hour in culture. Afterwards cells were washed and stained for the expression of CD3 (anti-CD3-BV421, clone 17A2; BioLegend), CD4 (anti-CD4-APC-Fire, clone RM4-5; BioLegend), CD8 (anti-CD8-FITC, clone 53-6.7; BioLegend), CD19 (anti-CD19-PerCP Cy5.5, clone 6D5; BioLegend), CD25 (anti-CD25-PerCP Cy5.5, clone PC61; BioLegend), and living cells using L/D Aqua in BV510 (BioLegend) for 40 min at 4°C. Further, cells were prepared for intracellular staining using the Fixation/Permeabilization solution kit (Thermo Fisher Scientific). Antibodies for Foxp3 (anti-Foxp3 AF647, clone FJK-16s; eBioscience), IFNγ (anti-IFNγ-PE-Cy7, clone XMG1.2; BD), and IL-17A (anti-IL-17A-AF647, clone TC11-18H10.1; BioLegend) were diluted in permbuffer (Thermo Fisher Scientific) and applied to the cells for 30 min at 4°C. The flow cytometric analyses were performed using a cytofluorometer (FACS Canto II, BD) and data were evaluated using FCS-express 5 (De Novo Software).

### RNA-Isolation and cDNA Synthesis

Total RNA was isolated from inguinal LN and synovial cells at the end of AIA using the RNeasy Mini Kit (Qiagen). cDNA synthesis was performed according to First Strand cDNA Synthesis Kit manual (Thermo Fisher Scientific). The PCR mixture contained 10 μl SsoAdvanced Universal SYBR Green Supermix (Biorad), 1 μl of 5′ and 3′ primer (forward and reverse, each at 0.2 μM), and 5 μl RNA reverse transcription product corresponding 12.5 ng of cDNA. The reaction was adjusted to 20 μl with aqua ad iniectabilia (Braun). After an initial denaturation step at 95°C for 3 min, 45 cycles of denaturation (94°C for 15 s), annealing (61°C for 15 s), and extension (72°C for 15 s) were performed using Bio-Rad CFX96 Touch Real-Time PCR Detection System (Biorad). Gene expression was calculated relatively to the housekeeping gene *Rpl4* (Ribosomal Protein L4) or β*-actin* regarding osteoclast experiments. Primer sequences are listed in Supplemental Table 1.

### Histological Analyses and Arthritic Score

Bone tissue was fixed overnight in 10% formalin, decalcified for 2 h in Osteomoll (Merck) and embedded in paraffin. Paraffin sections (4 μm) were stained with Safranin O for the morphological evaluation of inflammatory cell influx, synovitis, cartilage degradation (proteoglycane content) and bone resorption. Disease severity was assessed using the OsteoMeasure Analyses System (Osteometrics). For histological TRAP-analyses bone samples were gently decalcified in EDTA solution (Teitel buffer) and stainings were performed using the leukocyte acid phosphatase kit 386A (Sigma-Aldrich), according to manufacturer's instructions.

### Bead Based Array for Cytokine Detection

Supernatants from mBSA-restimulated LN and synovial cells were used to determine the cytokine expression profiles, using the LEGENDplex™ Mouse Inflammation Panel (13-plex) cytometric bead array (BioLegend), according to manufacturer's protocol.

### TGF-β ELISA

The TGF-β levels in the supernatants of mBSA-restimulated synovial cells were determined by ELISA according to manufacturer's instructions **(**TGF beta-1 Human/Mouse Uncoated ELISA Kit; Thermo Fisher Scientific).

### RANK L-ELISA

RANKL concentrations in sera from day 10 AIA mice were assessed using the TRANCE/RANK L/TNFSF11 Quantikine ELISA Kit (R&D Systems), according to manufacturer's instructions.

### HPLC-Analysis of Amino Acid metabolites

The amino acid assay is based on the derivatization of the amino acids in the sample by using phenyl isothiocyanate in the presence of isotope-labeled internal standards using liquid chromatography (LC-) MS/MS. Separation and quantitation of the resulting phenylthiocarbamyl derivatives is done by reverse-phase liquid chromatography coupled with an MS/MS-system for selective detection in MRM mode (API 6500 Q Trap; Sciex, Framingham, USA).

Briefly: All wells of a V-bottom microplate made from polypropylene were preconditioned using 10 μl of 0.2 M NaHCO3. The plate was allowed to dry. Samples (10 μl) and internal standard solution (10 μl) were pipetted into the cavities of the microplate. Then the liquid in the cavities was dried under nitrogen air flow. The dried samples were wetted with 25 μl of derivatization buffer (made of equal parts of pyridine, ethanol and water) and dried again. After that 25 μl of a freshly prepared solution of derivatization buffer and PITC (5%) was added to the dried wells and allowed to react for 30 min on a shaker. Following another drying step, the remaining substance in the wells was dissolved in 250 μl of an ammonium acetate solution prepared with methanol. Two-hundred microliter of this liquid were transferred to a deep well plate, and mixed with water (200 μl). After short centrifugation the prepared samples were put into the auto-sampler of our LC-MS/MS-System and measured. Separation and quantitation of the resulting phenylthiocarbamyl derivatives was done by reverse-phase liquid chromatography coupled with an MS/MS-system for selective detection in MRM mode. For the liquid chromatography part an Agilent Eclipse XDB-C18 3.5 μm, 3 × 100 mm column was used as stationary phase. Mobile Phase was made up out of two solutions: 0.2% formic acid in water and 0.2% formic acid in acetonitrile running a gradient ramping toward higher acetonitrile content. The following transitions were used for measurement in MRM-mode (including the matching deuterated internal standards for evaluation): Kynurenin: 344.2/146.2, d6-Kynurenin 350.2/151.2, Trp 340.2/188.2, d8-Trp 348.2/195.2, Met 285.1/104.2, d3-Met 288.1/107.2.).

### Generation of Synovial Fibroblast and DC Cultures

Hind limbs were removed from healthy C57BL/6 mice and cleared from residual tissue. The synovial area was digested in 2 mg/ml collagenase I solution (Merck) on a shaking incubator (2 h, 37°C, 1,200 rpm). The cell suspension was filtered through a 70 μm strainer, centrifuged (5 min; 4°C; 1,200 rpm) and the pellet resuspended and cultured in 10 ml fibroblast culture medium (RPMI 1640 (Lonza), supplemented with 100 U/ml penicillin, 100 μg/ml streptomycin, 2 mM L-glutamine (L-Glutamine–Penicillin–Streptomycin solution; Sigma-Aldrich), in the presence of 10% heat-inactivated FCS (Merck) and 0.2% amphotericin B (Gibco), in 25 cm^2^ cell culture flasks (Thermo Fisher Scientific). After passage 3, cells were transferred into 24 well plates (Falcon) until a confluency of 80–90% was reached. Cells were incubated with sCD83 (25 μg/ml), or stimulated with 100 U/ml IFNγ (PeproTech) as a positive control, and cultures were harvested at indicated time points for RNA analyses. DCs were generated as described before (22). Incubation with sCD83 was performed analogously to fibroblast culture. TGFβ (BioLegend) was used as positive control in a concentration of 10 ng/ml.

### Statistical Analysis

All data are expressed as mean ± SEM. Statistical significance was calculated using Student's *t*-tests for single comparison or the Mann-Whitney U test for nonparametric distribution. Grouped data were analyzed using One- or Two-way ANOVA. All calculations were performed using GraphPad Prism 7 (GraphPad). *P*-values < 0.05 were considered significant.

## Results

### sCD83 Ameliorates Disease Severity in Murine Arthritis

To investigate the modulatory effect of sCD83 *in vivo*, murine AIA was established in 8 weeks old mice and sCD83 was applied during the immunization phase, as depicted in Figure 1A. The sCD83 treated group showed significantly reduced joint swelling at peak of disease and an accelerated resolution of inflammation, compared to mock controls (Figure 1B). Also histological disease severity and joint destruction were significantly reduced in sCD83 treated animals as shown by the decreased frequency of large osteoclasts and Safranin O stainings of the knee joints (Figures 1C,D). The total arthritic score, which is a morphological assessment of synovitis, cartilage degradation and bone resorption, showed a significantly attenuated disease progression in sCD83 treated animals (Figure 1E). Digital histomorphometric quantification of tissue using OsteoMeasure demonstrated lower levels of cartilage destruction (Figure 1F) as well as an up to 5-fold reduced inflammatory area (Figure 1G), in sCD83 treated mice compared to mock controls. When cells from the inguinal LN were isolated at the end of the experiment and inoculated with mBSA for 72 h to induce antigen specific T cell proliferation, re-stimulated cells derived from sCD83 treated mice showed a highly significant reduced proliferation rate upon antigen specific mBSA stimulation in a concentration dependent manner (Figure 1H).

Disease modulation by sCD83 was also assessed in a second arthritis model, i.e., the STA. Interestingly, also in this model, sCD83 treated mice revealed a reduced clinical score upon daily sCD83-administration over a period of 15 days (day −1–13) (Supplemental Figure 1A). Even after a second K/BxN serum-transfer on day 31, without any additional sCD83 application, arthritic symptoms were reduced in sCD83 mice (Supplemental Figure 1B). Histological analysis of sCD83-treated mice demonstrated strongly reduced numbers of inflammatory cells (HE) as well as an enhanced protection from cartilage destruction and bone resorption in comparison to mock controls (Supplemental Figure 1C).

### sCD83 Modulates Cytokine Expression Within the Synovium of AIA Mice and Influences Osteoclastogenesis *in vitro*

**Figure 2:**
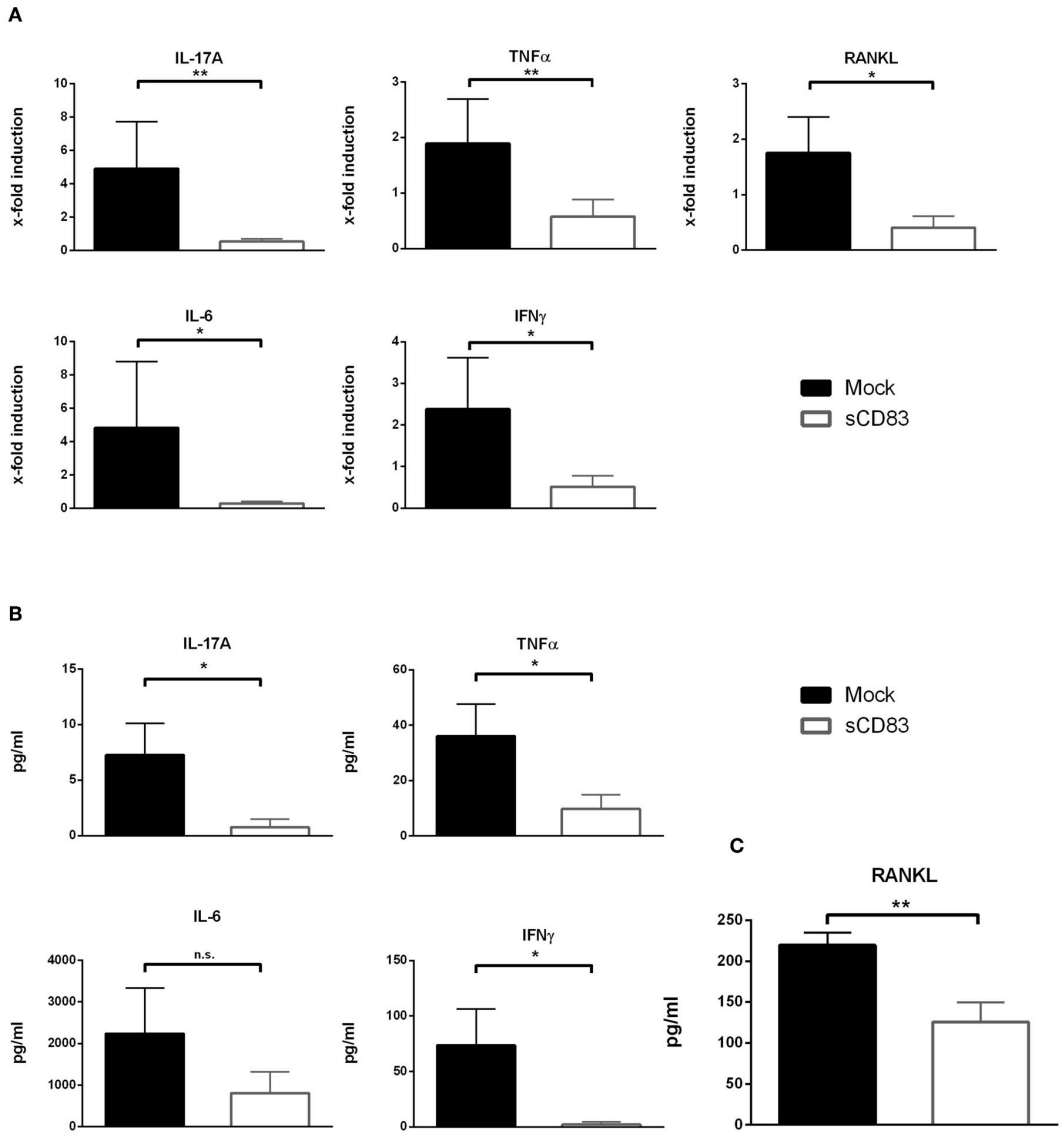
sCD83 inhibits the expression of pro-inflammatory cytokines within the synovium. **(A)** qPCR analyses of synovial RNA expression levels for arthritis related genes. *Rpl4* was used as housekeeping gene (sCD83 *n* = 8, mock *n* = 5). **(B)** Supernatants of synovial cell culture were analyzed using CBA method (sCD83 *n* = 4, mock *n* = 4). **(C)** Concentrations of RANKL were determined in day 10 serum samples by ELISA (sCD83 *n* = 10, mock *n* = 11) Data are illustrated as mean ± SEM and statistically analyzed using **(A,B)** student's *t* test and **(C)** Mann-Whitney test. Asterisks mark statistically significant difference (^*^p < 0.05 and ^**^p < 0.01). The absence of asterisks indicates that there is no statistical significance.

To gain further insights into the local molecular mechanisms, RT-PCR analyses of the synovial tissue, analyzing the expression of inflammatory transcripts associated with arthritis, were performed as shown in Figure 2. Within the synovium of sCD83 treated mice the expression levels of IL-17A, IFNγ, IL-6, and TNFα were significantly reduced (Figure 2A). Moreover, expression of RANKL, which is an essential cytokine for osteoclastogenesis (23), was significantly downregulated in sCD83 treated mice. These results were also confirmed on protein level, using CBA analyses of supernatants derived from cultured synovial cells (Figure 2B). Moreover, significantly reduced concentrations of RANKL were detected in sera derived from sCD83 treated mice (Figure 2C).

**Figure 3:**
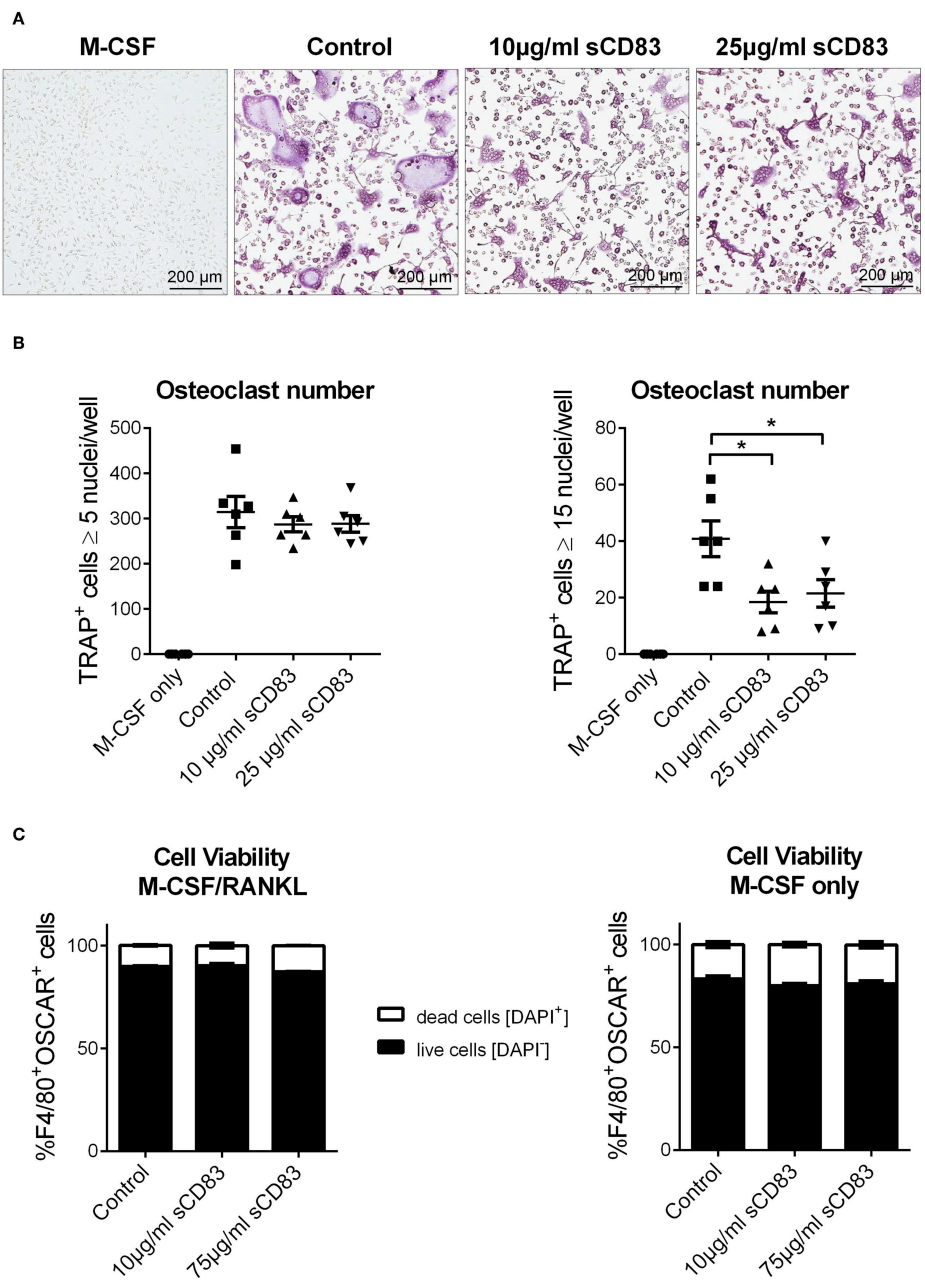
sCD83 modulates cellular fusion of multinucleated osteoclasts. Osteoclast differentiation of bone marrow mononuclear cells treated with MCSF and RANKL in the presence of different sCD83 concentrations. Osteoclasts were identified as multinucleated tartrate resistant acid phosphatase positive cells (purple). **(A)** Microphotographs showing osteoclasts. **(B)** Quantification of small (left) and large (right) osteoclasts with *n* = 6. **(C)** Flow cytometric analysis for DAPI^+^ osteoclasts (*n* = 3). Data are illustrated as mean ± SEM. **(B)** One-Way ANOVA. Asterisks mark statistically significant difference (^*^p < 0.05). The absence of asterisks indicates that there is no statistical significance.

**Figure 4:**
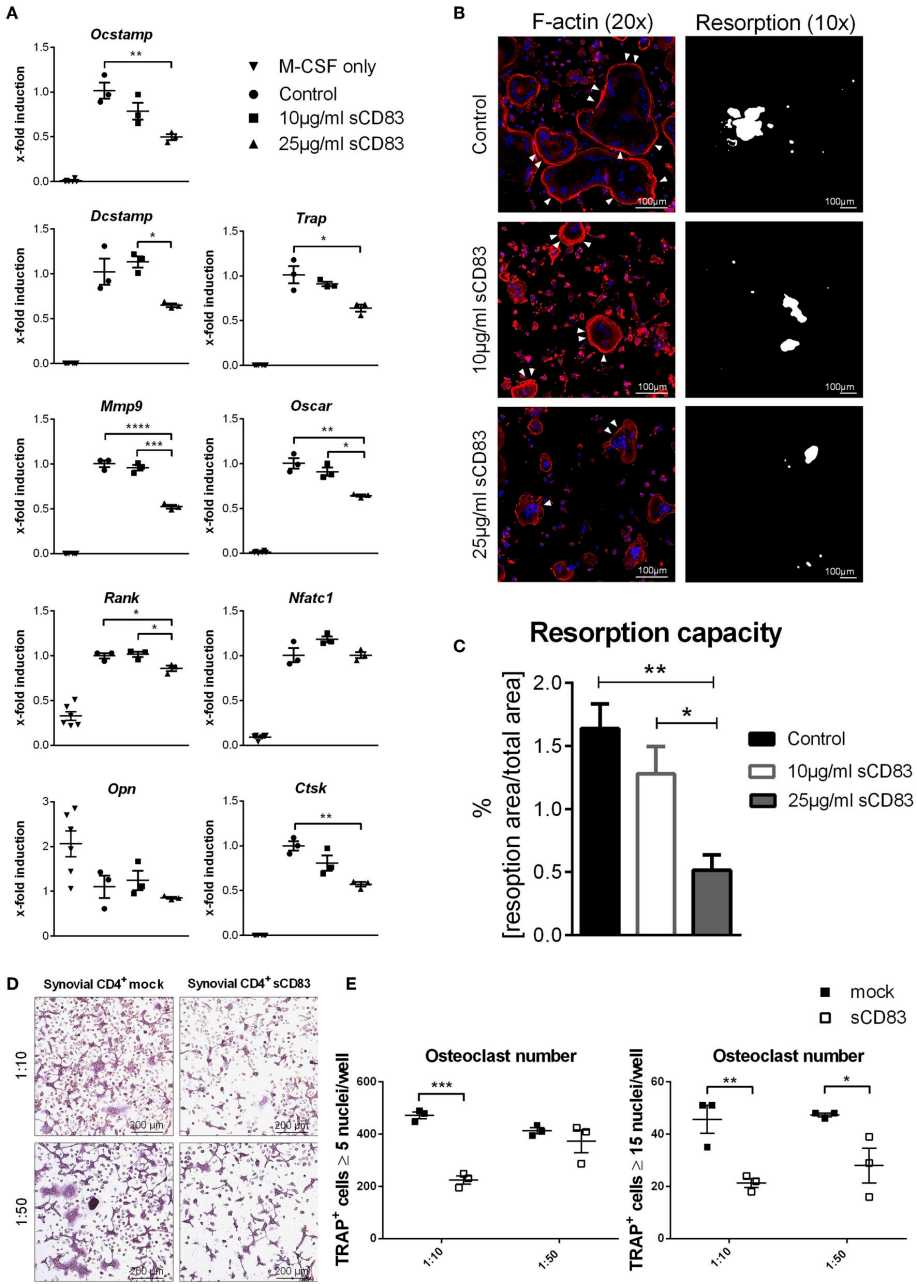
Osteoclastogenesis and their function is impaired in the presence of sCD83. **(A)** RT-PCR analyses regarding the expression of osteoclast related genes (*n* = 3). **(B)** Representative pictures of fluorescent F-actin stainings (20 × magnification) and resorption assays (10 × magnification), from osteoclasts generated in the presence of sCD83. **(C)** Resorption activity data (*n* ≥ 4). **(D)** Representative images used for the quantification of small (**E**, left) and large (**E**, right) multinucleated tartrate resistant acid phosphatase positive cells, which were co-cultured with synovial CD4^+^ cells derived from mock- or sCD83-treated mice (*n* = 3). Data are illustrated as mean ± SEM. One-Way ANOVA. Asterisks mark statistically significant difference (^*^p < 0.05, ^**^p < 0.01, ^***^p < 0.001, and ^****^p < 0.0001). The absence of asterisks indicates that there is no statistical significance.

In addition, bone marrow cells were isolated and subjected to *in vitro* osteoclast differentiation. Different concentrations of sCD83 or PBS were added and TRAP-staining was performed on day 5 to visualize osteoclast formation. The formation of large osteoclasts, containing more than 15 nuclei per cell, was clearly diminished by sCD83 (Figures 3A,B). In order to exclude possible toxic effects of sCD83 during osteoclastogenesis, viability of osteoclast cultures was assessed by DAPI-staining and flow cytometric analyses. Even at high sCD83 concentrations (up to 75 μg/ml), no toxic effects were observed when compared to control incubated cells (Figure 3C). qPCR analyses of osteoclast related genes, cultured in the presence of sCD83, revealed a significant, and concentration dependent downregulation of transcripts associated with cell fusion (i.e., *DC-Stamp, OC-Stamp*) and bone resorption (i.e., *Cathepsin K, Mmp9, and Trap*). Additionally, the expression of RANK and Oscar was significantly reduced in the presence of 25 μg/ml sCD83, while no modulation was observed for Nfatc1 and Opn (Figure 4A). To elucidate the impact of sCD83 on osteoclast activity, F-actin staining and resorption assays were performed. Again, we observed an inhibitory effect of sCD83 on the formation of large osteoclasts and a significantly reduced resorption activity, which was further supported by a reduced F-actin ring formation, a crucial structure for osteoclast-activity(24) (Figures 4B,C). To further substantiate the link between the *in vivo* findings observed in the AIA model and the impact of sCD83 on osteoclast formation *in vitro*, osteoclastogenesis analyses were performed in the presence of synovial CD4^+^ lymphocytes, since T cells play a crucial role in the onset of AIA (25). Interestingly, cells which were cultured in the presence of synovial T cells, derived from sCD83-treated AIA mice, showed a strongly reduced number of multinuclear large osteoclasts compared to mock controls. Moreover, at the higher ratio, i.e., 1:10, synovial CD4^+^ T cells from sCD83-treated mice, not only hampered osteoclast fusion, but also osteoclast differentiation from precursor cells (Figures 4D,E).

### sCD83 Enhances Resolution of Inflammation Also in a Flare Up Reaction and Provides Antigen Specific Long Term Modulation of Inflammatory Immune Responses in Arthritis

**Figure 5:**
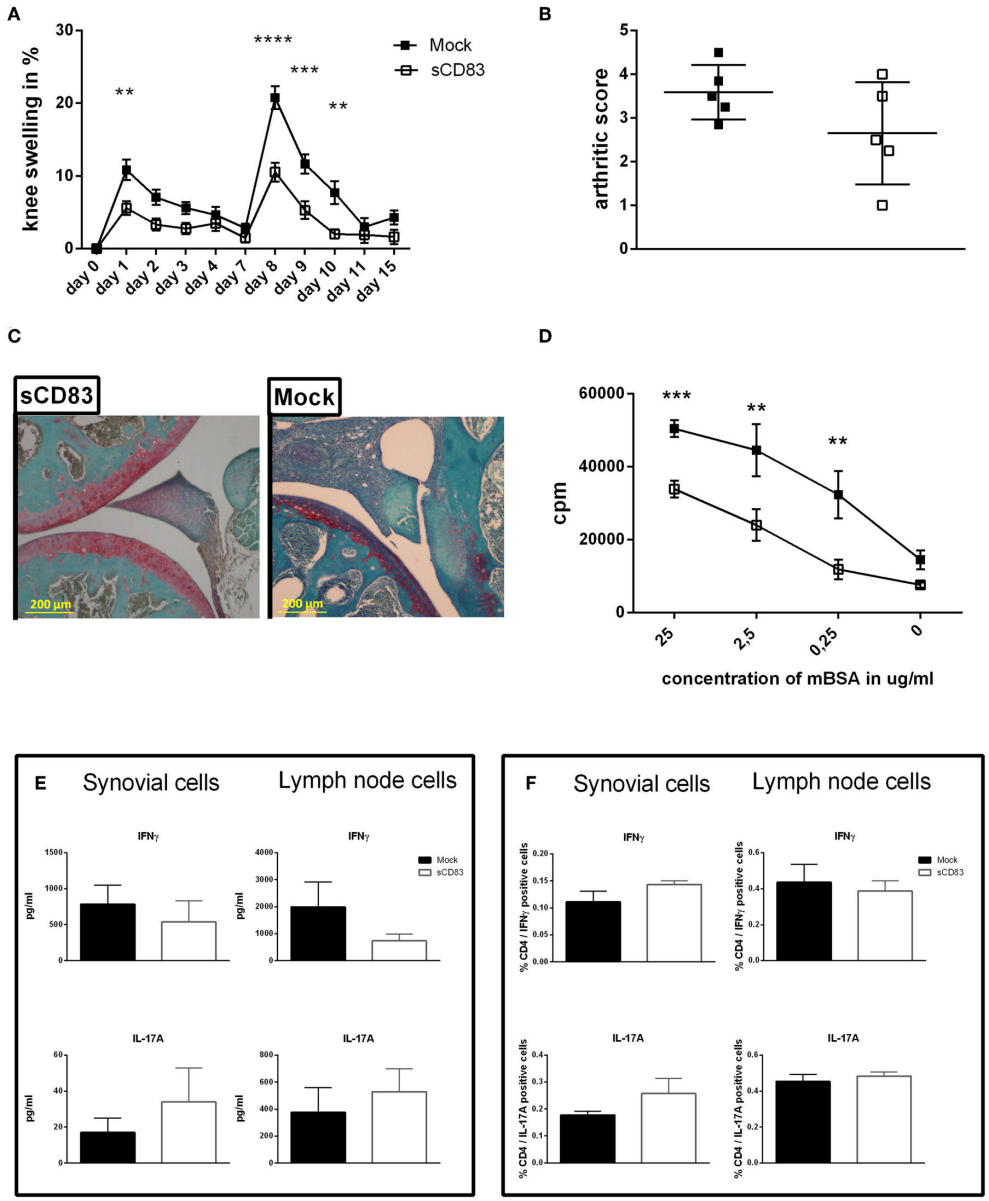
sCD83 induces long term and antigen specific protection from arthritis. A flare up reaction of antigen-induced arthritis (AIA) was induced by a second i.a. mBSA injection at day 7. **(A)** Percent increase of knee swelling (normalized to baseline) after the local i.a. injection of mBSA (sCD83 *n* = 10, mock *n* = 10). **(B)** Arthritic score based on cartilage degradation, bone resorption, and inflammation obtained from Safranin O stained knee joint sections (0 = normal and 5 = severe; sCD83 *n* = 5, mock *n* = 5). **(C)** Representative 4 μm Safranin O stained knee joint sections of sCD83 or mock treated mice at day 17. **(D)** antigen specific T cell proliferation of lymph LN cells from day 17 after mBSA stimulation, analyzed by radioactive tritium incorporation (sCD83 *n* = 9, mock *n* = 8). **(E)** IFNγ and IL-17A levels in supernatants from mBSA restimulated inguinal LN- (sCD83 *n* = 5, mock *n* = 4) and synovial cells (sCD83 *n* = 5, mock *n* = 5). **(F)** Flow cytometric analyses for intracellular IL-17A and IFNγ expression upon PMA and ionomycin stimulation for 6 h of inguinal LN- (sCD83 *n* = 9, mock *n* = 10) and synovial cells (sCD83 *n* = 4, mock *n* = 4). Data are illustrated as mean ± SEM. **(A,D)** Two way ANOVA and **(B,E,F)** Mann-Whitney test. Asterisks mark statistically significant difference (^**^p < 0.01, ^***^p < 0.001, and ^****^p < 0.0001). The absence of asterisks indicates that there is no statistical significance.

Rheumatoid arthritis is accompanied by relapse associated with swelling, pain, and inflammation. Hence, to investigate the long-term disease modulating effect of sCD83, a flare-up reaction was induced in the AIA-model (Figure 5). Thus, a second i.a. injection of mBSA was performed on day 7 (after the first mBSA i.a. injection), without any additional application of sCD83. Noteworthy, within 3 days, joint swelling was significantly resolved in the sCD83 treated group, while control animals showed typical AIA-associated symptoms for significantly longer time periods (Figure 5A). Histological analyses of the affected joints of sCD83 treated mice confirmed reduced synovitis and reduced degradation of cartilage as well as bone in comparison to control mice (Figure 5B). Representative histologies are shown in Figure 5C and Supplemental Figure 2. Further, mBSA-specific T cell proliferation of inguinal LN cells was reduced in sCD83 treated mice compared to mock controls (Figure 5D). mBSA-restimulated synovial and LN cells, derived from sCD83 treated mice showed reduced IFNγ levels, while IL-17A was not affected (Figure 5E). In contrary, equal IFNγ and IL-17A secretion levels were observed in sCD83- and mock treated mice after PMA- and ionomycin stimulation (Figure 5F; gating strategy see Supplemental Figure 3). These data indicate that sCD83 modulates antigen-specific T cell rather than broadly inhibiting T cell activation.

### Indoleamine 2,3-dioxygenase (IDO) Plays a Crucial Role in sCD83 Induced Resolution of Inflammation

**Figure 6:**
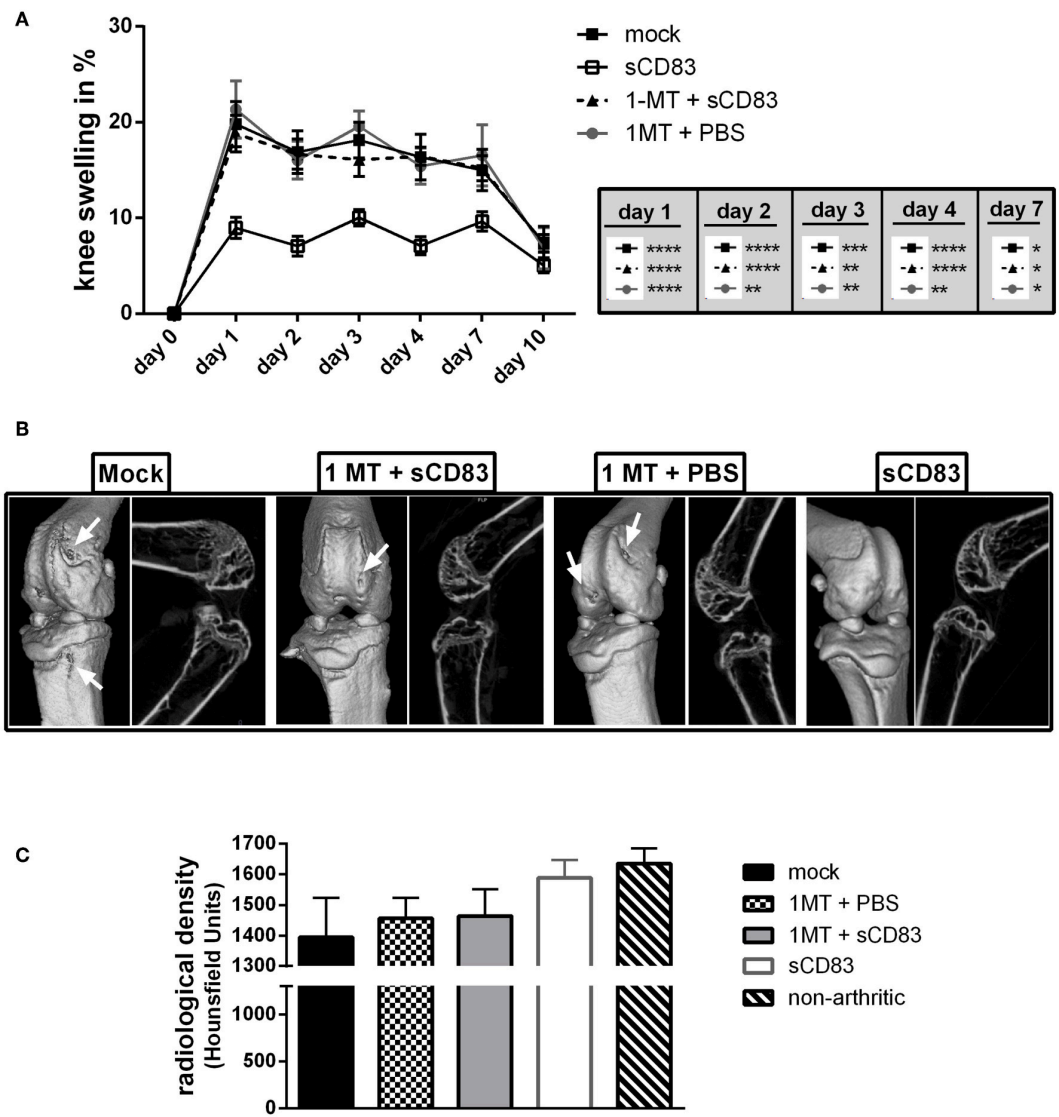
IDO plays a crucial role in sCD83 induced protection from bone destruction. To analyse the functional role of IDO in sCD83 induced regulatory mechanisms, 1-MT releasing pellets, which block IDO activity, were inserted s.c. at day −22 before the first application of sCD83. **(A)** Percent increase of knee swelling (normalized to baseline) after the local i.a. injection of mBSA (sCD83 *n* = 11, mock *n* = 10, 1-MT + sCD83 *n* = 11, 1-MT + PBS *n* = 8). **(B)** Representative μ-computed tomograpy images of knee joints. **(C)** Quantification of bone mass of femoral epiphysis (sCD83 *n* = 8, mock *n* = 10, 1-MT + sCD83 *n* = 10, 1-MT + PBS *n* = 6). Data are illustrated as mean ± SEM. **(A)** Two way ANOVA and **(C)** One-Way ANOVA. Asterisks mark statistically significant difference (^*^p < 0.05, ^**^p < 0.01, ^***^p < 0.001, and ^****^p < 0.0001). The absence of asterisks indicates that there is no statistical significance.

IDO is a key regulator of the T cell immune response and was described as a therapeutic target for RA therapy (26). Due to its enzymatic activity IDO is able to convert tryptophan, which is an essential amino acid for T cell proliferation and survival (27), into kynurenine. On the one hand tryptophan starvation leads to reduced T cell activation, while kynurenine itself on the other hand, enhances Treg induction/ expansion via the Ahr-signaling pathway (28, 29). Further, the signaling activity of IDO was shown to induce TGF-β which is crucial for Treg function (30) and long term tolerance induction (31). To elucidate the functional role of IDO in sCD83 induced mechanisms in arthritis the enzymatic activity of IDO was blocked by 1-MT (see Figure 6), which is a potent IDO inhibitor (32). Mice receiving sCD83 in the presence of 1-MT showed a progressive arthritis comparable to the mock or 1-MT + PBS treated animals (Figure 6A) (33). Furthermore, mock controls, 1-MT + PBS and 1-MT + sCD83 treated mice developed bone damage on day 10 after mBSA injection (Figure 6B, see white arrows in the representative μCT reconstructions). In sharp contrast, joints derived from sCD83 treated mice, showed almost no bone destruction (Figure 6B, right panel). In addition, mock as well as 1-MT treated controls developed severe loss at the femoral epiphysis (Figure 6C), while sCD83 treated mice revealed hardly any resorption of bone tissue and were comparable to samples derived from non-arthritic animals.

**Figure 7:**
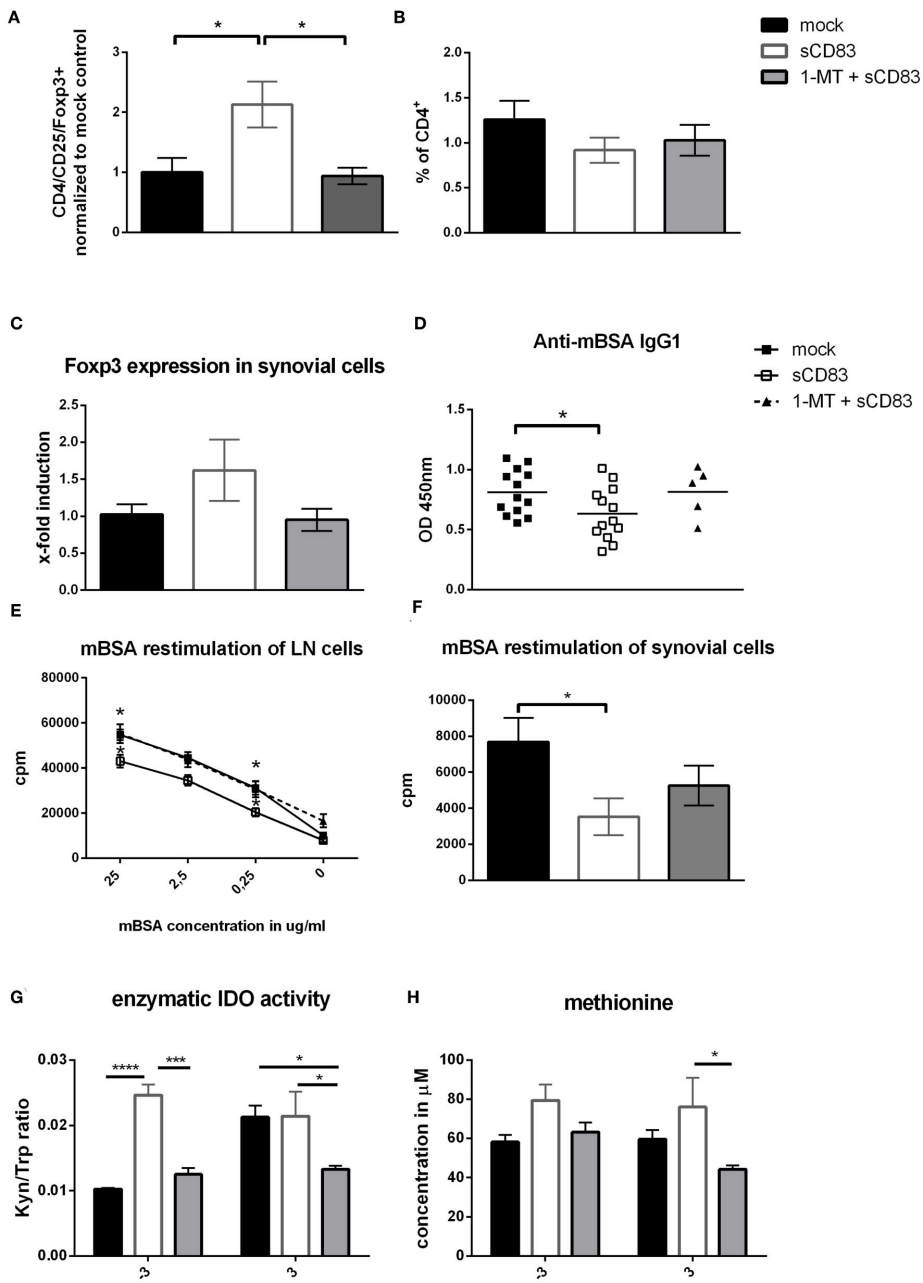
IDO plays a crucial role in the sCD83 mediated induction of Tregs within the synovium and regulates methionine balance. **(A–C)** CD4^+^ T cell as well as regulatory T cell numbers (CD4^+^, CD25^+^, and Foxp3^+^; pool from three independent experiments normalized to mock control) within the synovium assessed by flow cytometry (sCD83 *n* = 14, mock *n* = 13, 1-MT + sCD83 *n* = 9) and qPCR (sCD83 *n* = 4, mock *n* = 4, 1-MT + sCD83 *n* = 4). **(D)** Anti-mBSA specific IgG1 antibody levels in the sera of AIA mice on day 10 (sCD83 *n* = 13, mock *n* = 13, 1-MT + sCD83 *n* = 5). **(E)** Antigen specific T cell proliferation of inguinal LN cells upon mBSA restimulation analyzed by radioactive tritium incorporation (sCD83 *n* = 5, mock *n* = 5, 1-MT + sCD83 *n* = 10). **(F)** Antigen specific T cell proliferation of synovial cells after mBSA restimulation (sCD83 *n* = 4, mock *n* = 4, 1-MT + sCD83 *n* = 4). **(G)** Sera from AIA mice were collected and analyzed by HPLC to assess the kynurenine to tryptophan ratio and methionine concentration **(H)** (sCD83 *n* = 5, mock *n* = 5, 1-MT + sCD83 *n* = 5). Data are illustrated as mean ± SEM. Statistical analysis was performed using the One-way ANOVA **(A–D,F)** and Two way ANOVA **(E,G,H)**. Asterisks mark statistically significant difference (^*^p < 0.05, ^***^p < 0.001, and ^****^p < 0.0001). The absence of asterisks indicates that there is no statistical significance.

To gain further insights into the local regulatory mechanisms in the joints, synovial cells were isolated and analyzed by flow cytometry (Figure 7). Most notably regulatory T cells were significantly increased in their frequency after sCD83 treatment, but not in mock controls or mice treated with sCD83 but receiving the IDO inhibitor 1-MT (Figure 7A, representative FACS plots Supplemental Figure 4). The percentage of CD4^+^ T cells was similar in all different treatment groups (Figure 7B). These observations were confirmed by qPCR-analyses of synovial cells, where the sCD83 treated group showed an enhanced expression of Foxp3 (Figure 7C). In sharp contrast, application of 1-MT abrogated the sCD83 induced accumulation of Tregs.

Since antibodies play a crucial role in RA, we analyzed the presence of mBSA-specific IgG1 in the sera of AIA mice, and observed significantly reduced anti-mBSA-IgG1 levels in sCD83 treated mice on day 10 (Figure 7D). However, application of 1-MT completely reversed sCD83 mediated effects on anti-mBSA-IgG1 levels. mBSA restimulation of synovial and LN cells showed again reduced levels of antigen-specific T cell proliferation within the sCD83 treated group (Figures 7E,F). In contrast the proliferation rate of the 1-MT + sCD83 treated mice was comparable to mock controls, both for LN and synovial cells. To determine the enzymatic kinetics of IDO during AIA *in vivo*, sera were collected at different time points from different treatment groups and analyzed regarding to their tryptophan to kynurenine ratio, using HPLC. A strong and highly significant induction of IDO activity was observed in sCD83 treated mice especially on day −3 (Figure 7G), indicating the induction of an anti-inflammatory environment just before the onset of the effector phase. This upregulation of IDO activity was blocked by 1-MT. Moreover, and unexpectedly, at day −3 and +3 we observed significantly increased levels of methionine in the sCD83-, but not in mock- or 1-MT + sCD83 treated mice (Figure 7H). This is a very interesting finding, since methionine has been described to attenuate disease severity in arthritis (34).

**Figure 8:**
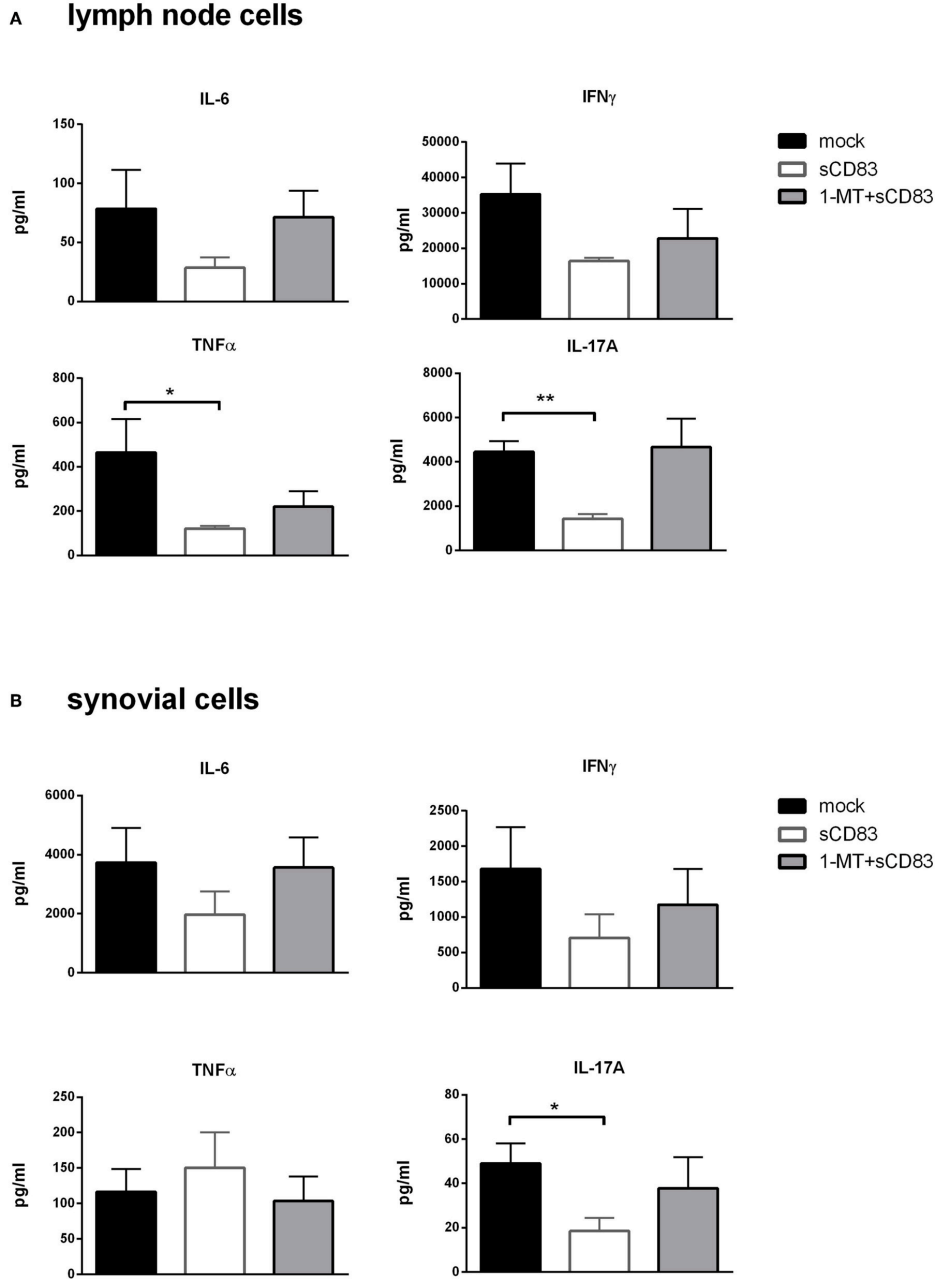
sCD83 induced inhibition of pro-inflammatory mediators is IDO dependent. **(A)** Cytokine production in mBSA restimulated inguinal LN cells (sCD83 *n* = 5, mock *n* = 5, 1-MT + sCD83 *n* = 5) and **(B)** synovial cells (sCD83 *n* = 4, mock *n* = 4, 1-MT + sCD83 *n* = 4). Data are illustrated as mean ± SEM. Mann-Whitney test. Asterisks mark statistically significant difference (^*^p < 0.05, ^**^p < 0.01,). The absence of asterisks indicates that there is no statistical significance.

Furthermore, analysis of cytokine secretion of these restimulated LN and synovial T cells showed that IL-6, IL-17A, TNFα, and IFNγ levels were strongly reduced in LN cells from sCD83 treated mice compared to mock controls or to those treated with 1-MT + sCD83 (Figure 8A). Likewise, in synovial cells production of IL-6, IL-17A, and IFNγ was reduced after sCD83 treatment (Figure 8B).

### TGF-β Is Mechanistically Involved in sCD83 Induced Modulation in Arthritis

**Figure 9:**
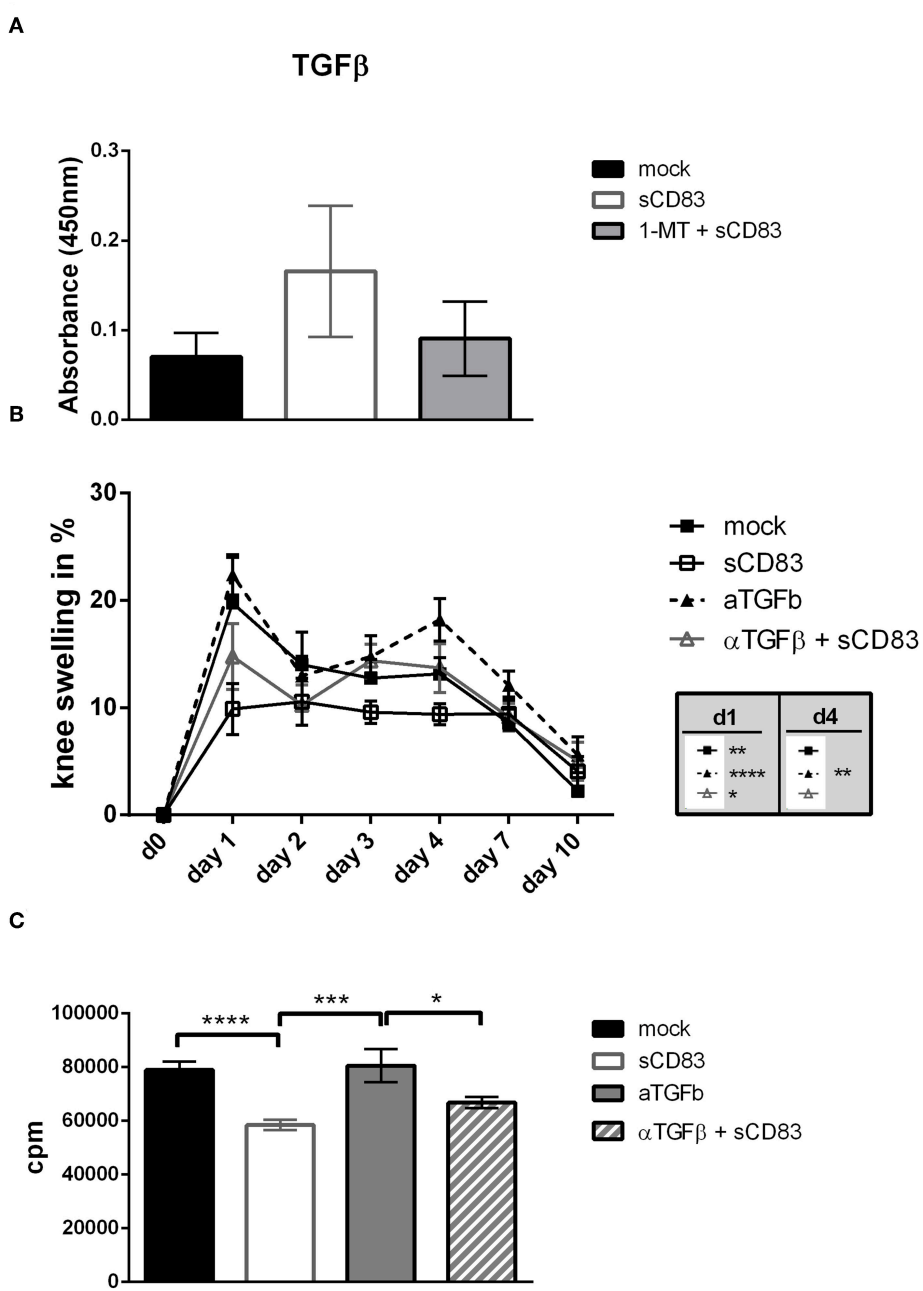
TGF-β is involved in sCD83 induced inhibition of arthritis. TGF-β was blocked by the systemic application of an anti-TGF-β antibody from day 0–9 and 20–28. **(A)** TGF-β levels in supernatants of mBSA restimulated synovial cells were determined by ELISA (sCD83 *n* = 4, mock *n* = 4, 1-MT + sCD83 *n* = 4). **(B)** Percent increase of knee swelling (normalized to baseline) after the local i.a. injection of mBSA (sCD83 *n* = 5, mock *n* = 5, α-TGF-β *n* = 5, α-TGF-β + sCD83 *n* = 5). **(C)** Antigen specific T cell proliferation of LN cells upon mBSA stimulation analyzed by tritium incorporation (sCD83 *n* = 5, mock *n* = 5, α-TGF-β *n* = 3, α-TGF-β + sCD83 *n* = 4). Data are illustrated as mean ± SEM. **(B)** Two way ANOVA and **(A,C)** One-Way ANOVA. Asterisks mark statistically significant difference (^*^p < 0.05, ^**^p < 0.01, ^***^p < 0.001, and ^****^p < 0.0001). The absence of asterisks indicates that there is no statistical significance.

Since TGF-β is an important mediator in Treg cell function we analyzed whether sCD83 treatment induces TGF-β. In synovial cell cultures from sCD83 treated arthritic mice we detected increased levels of TGF-β (Figure 9A), supporting a functional implication of the TGF-β-IDO pathway in sCD83 mediated resolution of inflammation. We therefore investigated, whether TGF-β plays a mechanistic role in sCD83 induced immune modulation. Hence, TGF-β activity was blocked *in vivo* by the daily injection of anti-TGF-β antibody during immunization phase (i.e., day −21 until day −12) and effector phase (i.e., day −1 until day 7) (33). Mice which received the anti-TGF-β antibody alone, showed a slightly but not significant increased joint swelling, compared to mock-treated mice (Figure 9B). However, in the presence of anti-TGF-β, the proresolving effect of sCD83 was partially abolished. Hence, the degree of arthritis in the sCD83/TGF-β treated group was between the one of sCD83 treatment and mock treated mice, indicating that TGF-β plays a role but is not exclusively responsible for sCD83 mediated anti-arthritic effects. This finding is in line with previous data that suggested that TGF-β induces IDO mediated long-term tolerogenic effects (31).

When assessing the effect of TGF-β inhibition on LN cell proliferation we found a slightly increased T cell proliferation upon mBSA-restimulation *in vitro*, when TGF-β was inhibited, (Figure 9C) supporting the *in vivo* data.

## Discussion

The immune-modulatory potential of sCD83 has been described in different autoimmune (13, 35) and transplantation models (10, 14). However, no data were available regarding arthritis, even though increased levels of sCD83 have been observed in the synovial fluid of RA patients (17, 36). Thus, in the present study we investigated the immune-modulatory properties of sCD83 in the AIA-model. We show that sCD83 has anti-inflammatory properties in arthritis and induces resolution of inflammation. This effect critically depends on sCD83 induced IDO activation, in conjunction with TGF-β expression. Application of sCD83 not only reduced the clinical symptoms of arthritis, but also inhibited the antigen-specific T cell proliferation upon mBSA-restimulation and impaired production of key inflammatory effectors, including IL-17A, TNFα, IFNγ, and IL-6. In accordance with these immune regulatory effects, joint destruction was also strongly reduced in sCD83 treated mice. Noteworthy, also RANKL expression, a key mediator for osteoclast differentiation, was strongly reduced in the synovium of sCD83 receiving mice, which explains the observed attenuated destruction of cartilage and bone. Thus, sCD83 obviously has the potential to inhibit bone destruction by osteoclasts in arthritis. In support of this hypothesis, *in vitro* osteoclastogenesis was impaired by sCD83 in a concentration dependent manner. qPCR analyses revealed a downregulated expression of osteoclast-fusion and bone resorption related genes in the presence of sCD83, which most likely contributes to the observed impaired phenotype. Thus, sCD83 does not directly modulate the differentiation of osteoclasts, but rather interferes with osteoclast fusion and activity. Recently, Horvatinovitch et al. reported the TLR4/MD2-complex as a receptor for sCD83 on monocytes (37). Since monocytes, which differentiate into osteoclasts (38) still maintain the expression of TLR4 on their surface (39), it is conceivable that sCD83 may modulate osteoclastogenesis via this pathway. Therefore, increased sCD83 concentrations, as detected in the synovial fluids of RA patients (17), might contribute to temporary resolution of arthritic symptoms and hamper osteoclast formation and activity. Furthermore, we observed an interesting modulatory effect of sCD83 on F-actin ring formation in mature osteoclasts. In 2004 Kotzor et al. described sCD83 mediated changes in the cytoskeleton of DCs which subsequently hampered DC clustering and their stimulatory activity (40). Thus, sCD83 might induce an impaired osteoclast phenotype by the modulation of the cell architecture and subsequently their activity.

In flare-up experiments for arthritis, which were performed by a second mBSA injection without additional sCD83 exposure, mice which received sCD83 only during the first mBSA injection were still maintaining better control of arthritis and faster resolution. This finding of prolonged tolerance reiterates previous data reporting sCD83 mediated long-term induction of regulatory mechanisms in the experimental-autoimmune-encephalitis (EAE) model (13). Furthermore, these data indicated, that such sCD83 induced effect is antigen-specific, since restimulation of LN cells, derived from sCD83 treated animals, with mBSA showed reduced IFNγ secretion, while there was no difference in cytokine expression after PMA/ionomycin stimulation. These results are in agreement with previous reports regarding heart and cornea transplantation experiments, also showing an antigen-specific immune modulation (10, 14).

Interestingly, the protective effect of sCD83 was completely abrogated in the presence of the IDO inhibitor 1-MT. We thus postulate that the enzymatic IDO activity is crucial for the sCD83 mediated effects. Induction of IDO via the application of sCD83 and its interaction with DC, T cells and osteoclasts marks the induction of regulatory pathways. IDO expression in myeloid cells has been shown to induce the generation of regulatory DCs, to inhibit differentiation of osteoclasts (41) and, as shown in this work, to inhibit proinflammatory cytokine production by LN and synovial cells. Analysis of bone structure in arthritis confirmed the crucial role of IDO, since the beneficial effects of sCD83 regarding bone destruction were abrogated when IDO was inhibited by 1-MT.

Further, IDO is a potent modulator of T cell immunity by two means. On the one hand IDO induces the enzymatic conversion of tryptophan into kynurenine (42). Tryptophan starvation induces an inhibitory effect on T cell proliferation (43), while on the other hand kynurenine potently induces regulatory T cells (44). In agreement with these reports we observed a highly increased kynurenine/tryptophan ratio in sCD83 treated mice, thereby inducing an anti-inflammatory environment shortly before the onset of the effector phase. Within the synovial cavities, synovial fibroblasts possess strong IDO activity (45), which could be induced upon sCD83 stimulation (Supplemental Figure 5) and therefore these cells may be responsible for the observed increased concentrations of sCD83 in the synovial fluids of RA patients (17).

Furthermore, an increased frequency of Foxp3^+^ regulatory T cells was found in the synovium of sCD83 treated mice, which was again IDO dependent since 1-MT reversed this sCD83 effect (Figure 5B). Tregs are important players during resolution of inflammation, which can be mediated via the expression of soluble factors such as TGF-β and IL10 (46, 47), the suppression of effector T cells (48) or metabolic modulation, via e.g., IL-2 deprivation (49). In addition to its enzymatic properties, IDO possesses also a signaling activity which promotes TGF-β expression, thereby enhancing the differentiation of regulatory T cells and finally the induction of long term tolerance mechanisms (28, 50).

Treg cells have been reported to regulate osteoclastogenesis e.g., via the secretion of TGF-β (46) and furthermore protect from bone destruction in arthritis (16). As described above in sCD83 treated animals we observed an increase in TGF-β production and enhanced numbers of Foxp3^+^ regulatory T cells, which correlated with reduced bone and cartilage destruction in AIA, extending previous observations (16, 46). In addition it has been reported that Tregs regulate osteoclast differentiation, in a CTLA-4 and IDO-dependent manner (41). Since sCD83 induces IDO expression and subsequently increases Treg numbers, it is very likely that this represents the underlying mechanism by which sCD83 inhibits bone and cartilage destruction in addition to the induction of resolution of inflammation. Additionally, when osteoclastogenesis was performed in the presence of synovial CD4^+^ T lymphocytes, derived from sCD83 or mock treated AIA mice, osteoclast formation was dramatically reduced in the sCD83 group. Since no additional sCD83 was added to these cultures, our results emphasize the role of a Treg mediated inhibitory environmental and/or direct effects on osteoclast formation within the synovia of sCD83-treated mice.

Interestingly, sCD83 induced the expression of TGF-β in an IDO dependent manner. To define the functional role of TGF-β in sCD83 mediated effects in arthritis we blocked TGF-β activity *in vivo*, using an inhibitory anti-TGF-β antibody. Noteworthy, blockade of TGF-β partially reversed the anti-arthritic effects of sCD83. Similar results were also observed by others, where the application of anti TGF-β antibodies resulted in increased AIA severity (33). These data indicate, that not only IDO but also TGF-β plays a role in sCD83 induced regulatory mechanisms in arthritis, and that both may exert synergistic effects. Thus, we hypothesize, that sCD83 induces TGF-β expression, which subsequently increases IDO levels and long term regulatory mechanisms, associated with resolution of inflammation.

An unexpected finding was the increase in the levels of the essential amino acid methionine in sCD83 treated mice. Since methionine cannot be synthetized *de novo*, sCD83 may influence the conversion of homocysteine to methionine, as it has been described previously (51). Increased levels of homocysteine are pro-inflammatory and associated with cardiovascular mortality in RA patients (52). A sCD83 induced shift toward methionine synthesis would therefore provide anti-inflammatory properties in RA (53). Also, methionine accumulation was IDO-dependent, since in the presence of 1-MT this effect was abrogated.

**Figure 10:**
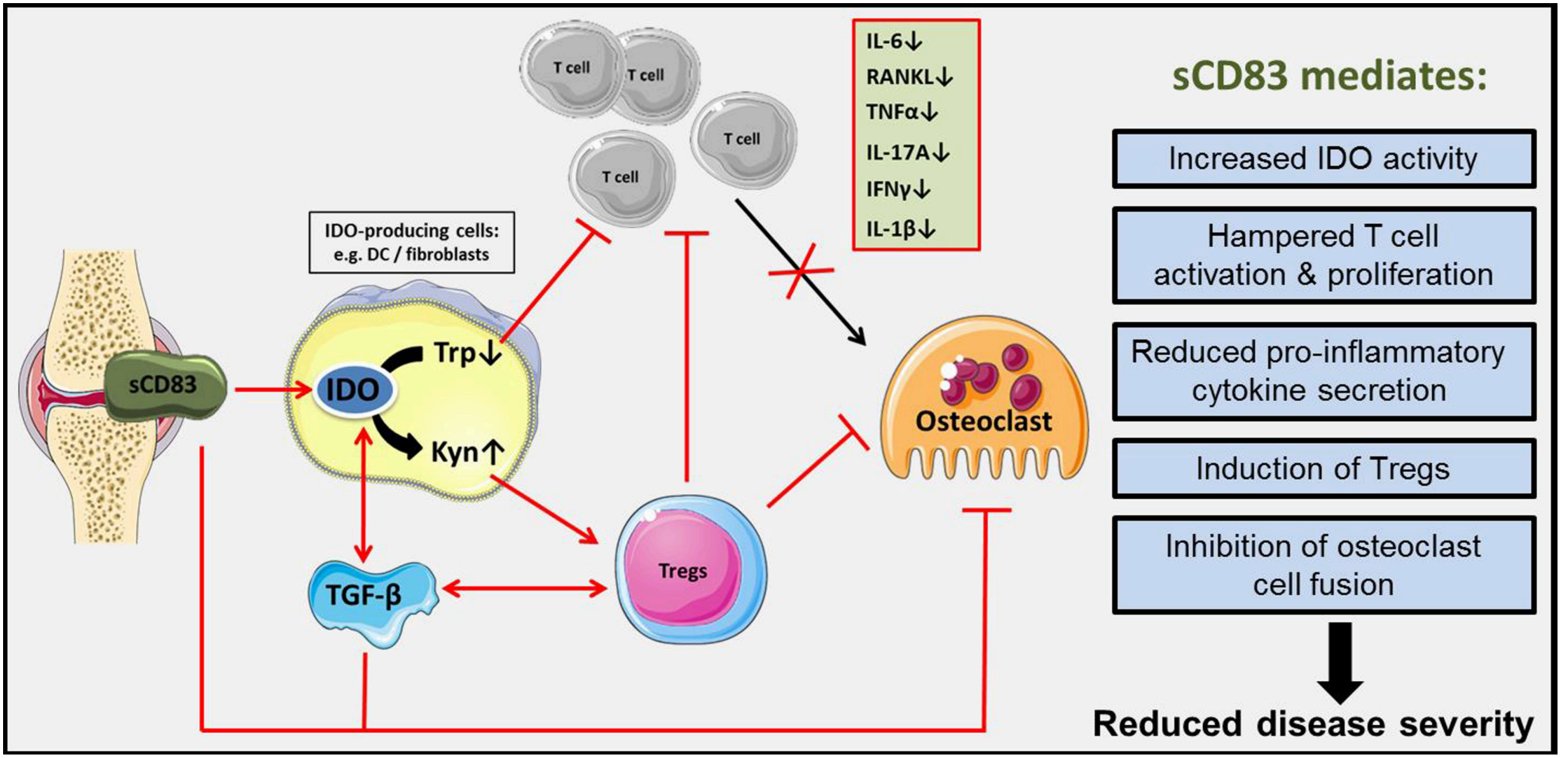
Schematic representation of sCD83 induced immune modulation in arthritis. sCD83 is expressed in arthritic joints and accumulates within the synovial fluid. There, sCD83 induces the enzymatic activity of IDO in responder cells (e.g., DCs, fibroblasts) enhancing the tryptophan (Trp) to kynurenine (Kyn) conversion. Tryptophan starvation induces an inhibitory effect on T cell proliferation, while on the other hand kynurenine potently induces regulatory T cells. In addition, sCD83 induces TGF-β expression and thereby long lasting IDO mediated Treg differentiation. Thus, we hypothesize, that sCD83 induces long term regulatory mechanisms, associated with resolution of inflammation and inhibition of bone destruction and cartilage damage in arthritis.

Taken together, sCD83 leads to a long-term and antigen-specific modulation of the immune response in arthritis (summarized in Figure 10). IDO and TGF-β play a crucial role in sCD83 induced reduction of inflammation and joint destruction in arthritis. Regulatory T cells, which are induced via a sCD83-IDO dependent mechanism, are essential for resolution of inflammation and long-lasting tolerogenic effects. Therefore, sCD83 provides an interesting candidate for inducing long-term control of the immune response and resolution of inflammation in RA.

## Ethics Statement

Animal housing and experimental studies were approved by local authorities in Würzburg.

## Author Contributions

EZ and AS conceived and designed the study, supervised experiments, and wrote the manuscript. DR and EZ performed and analyzed the majority of experiments. DR also prepared the manuscript. DA and AB performed and analyzed osteoclastogenesis experiments, quantified histological samples, and edited the manuscript. MR conducted HPLC and metabolite analyses. TB, SE, and MK performed micro-CT and radiological analyses. LS performed some experiments and analyzed the corresponding data. KP was responsible for DC cultures and the analyses of IDO expression. LB provided αTGF-ß antibody and edited the manuscript. GS provided scientific insight and edited the manuscript.

### Conflict of Interest Statement

LB is employed by Bioceros. The remaining authors declare that the research was conducted in the absence of any commercial or financial relationships that could be construed as a potential conflict of interest.

## Abbreviations

AIA: antigen -induced arthritis
BMM: bone marrow derived monocytes
CFA: complete Freund's adjuvant
cpm: counts per minute
DC: dendritic cells
IDO, indoleamine 2: 3-dioxygenase
IL: interleukin
IFNγ: interferon-γ
Kyn: Kynurenine
LN: lymph node
mBSA: methylated bovine serum albumin
PBS: phosphate-buffered saline
RA: rheumatoid arthritis
RANK/RANKL: receptor activator of nuclear factor kappa-B ligand
Rpl4: Ribosomal Protein L4
sCD83: soluble CD83
SEM: standard error of the mean
STA: serum transfer arthritis
TGF: transforming growth factor
TNFα: tumor necrosis factor α
Tregs: regulatory T cells
Trp: tryptophan.
