# Supplementary material for: Soluble CD83 Triggers Resolution of Arthritis and Sustained Inflammation Control in IDO Dependent Manner
Source: Front Immunol. 2019 Apr 2;10:633. doi: 10.3389/fimmu.2019.00633 (PMC6455294; doi:10.3389/fimmu.2019.00633)
Supplement: Supplementary file 1 [file Data_Sheet_1.pdf]

	Forward	Reverse
IL-17A	TCCAGAAGGCCCTCAGACTA	AGCATCTTCTCGACCCTGAA
FoxP3	CCCAGGAAAGACAGCAACCTT	CCTTGCCTTTCTCATCCAGGA
RANKL	CAGCCATTTGCACACCTCAC	CCCGATGTTTCATGATGCCG
TNF-alpha	GTGATCGGTCCCCAAAGGG	CCAGCTGCTCCTCCACTTG
IL-6	ACAAAGCCAGAGTCCTTCAGAG	GAGCATTGGAAATTGGGGTAGG
Rpl4	GCTGAACCCTTACGCCAAGA	TCTCGGATTTGGTTGCCAGT
IFNγ	GCTTTGCAGCTCTTCCTCAT	GTCACCATCCTTTTGCCAGT
Beta-actin	TGTCCACCTTCCAGCAGATGT	AGCTCAGTAACAGTCCGCCTAGA
Rank	TTGTGGCAGGGGACTTTAAC	ATTGTCATCCTGCCCTCAAC
Oscar	CACACACACCTGGCACCTAC	GAGACCATCAAAGGCAGAGC
Nfatc1	GGTGCCTTTTGCGAGCAGTATC	CGTATGGACCAGAATGTGACGG
Opn	TCCAATCGTCCCTACAGTCG	AGCTGACTTGACTCATGGCT
Dc-stamp	TGGAAGTTCACTTGAAACTACGTG	CTCGGTTTCCCGTCAGCCTCTCTC
Oc-stamp	TTGCTCCTGTCCTACAGTGC	GCCCTCAGTAACACAGCTCA
Trap	CGACCATTGTTAGCCACATACG	TCGTCCTGAAGATACTGCAGGTT
Cathepsin K	AGGGCCAACTCAAGAAGAAAACT	TGCCATAGCCCACCACCAACACT
Mmp9	GCTGACTACGATAAGGACGGCA	TAGTGGTGCAGGCAGAGTAGGA

Table 1. Primer used for qRT-PCR

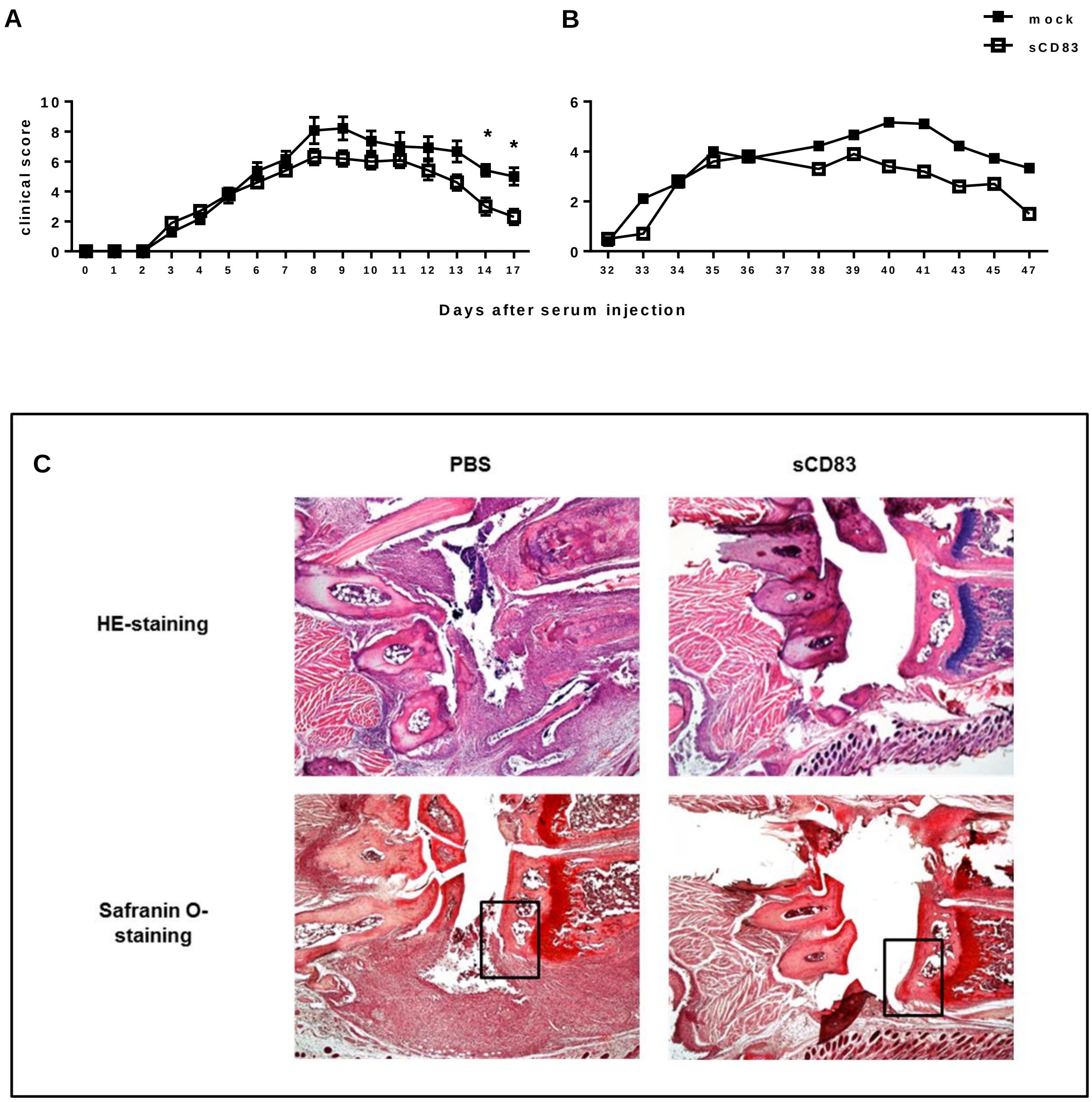

Suppl. Figure 1. sCD83 administration ameliorates the disease severity in serum transfer arthritis (STA) model and establishes a long term protection from flare up reaction. sCD83 treatment was performed by a daily i.p. injection of 100 µg sCD83 or PBS as mock control. STA was induced by the i.p. injection of K/BxN serum on day 0. (A) Daily scoring of the paw joint swelling after the initial STA-induction and (B) after the flare up reaction by a second K/BxN administration on day 31 without any additional sCD83 administration (sCD83 n = 5, mock n = 5). (C) Representative samples of HE and Safranin O stained PFA slides of the paw regions. Squares indicate cartilage loss and resorption of bone tissue. Data are illustrated as mean ± SEM. Asterisks mark statistically significant difference (*$p < 0.05$, **$p < 0.01$). Two way ANOVA analysis.

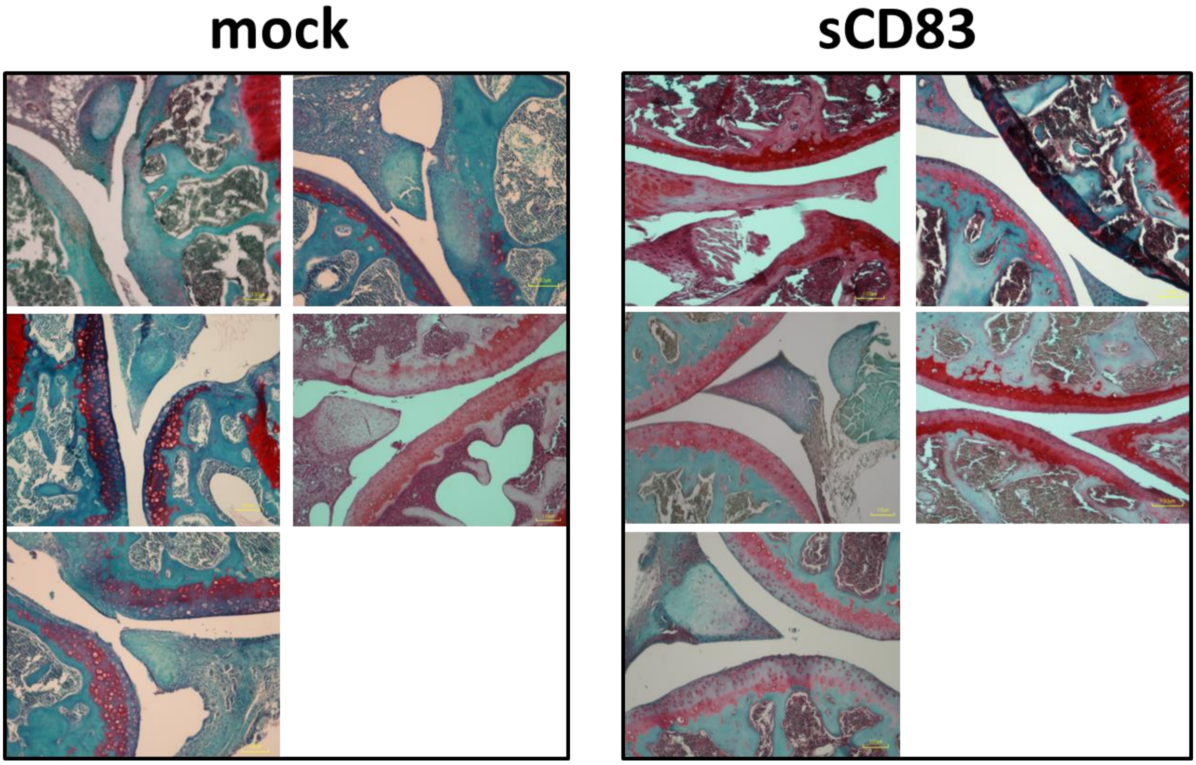

Suppl. Figure 2. Safranin O stainings from flared-up treated AIA mice. 4 µm paraffin slides were stained according to the histological Safranin O protocol to quantify arthritic disease severity. (Left) mock treated mice and (Right) sCD83 treated mice with each n = 5.

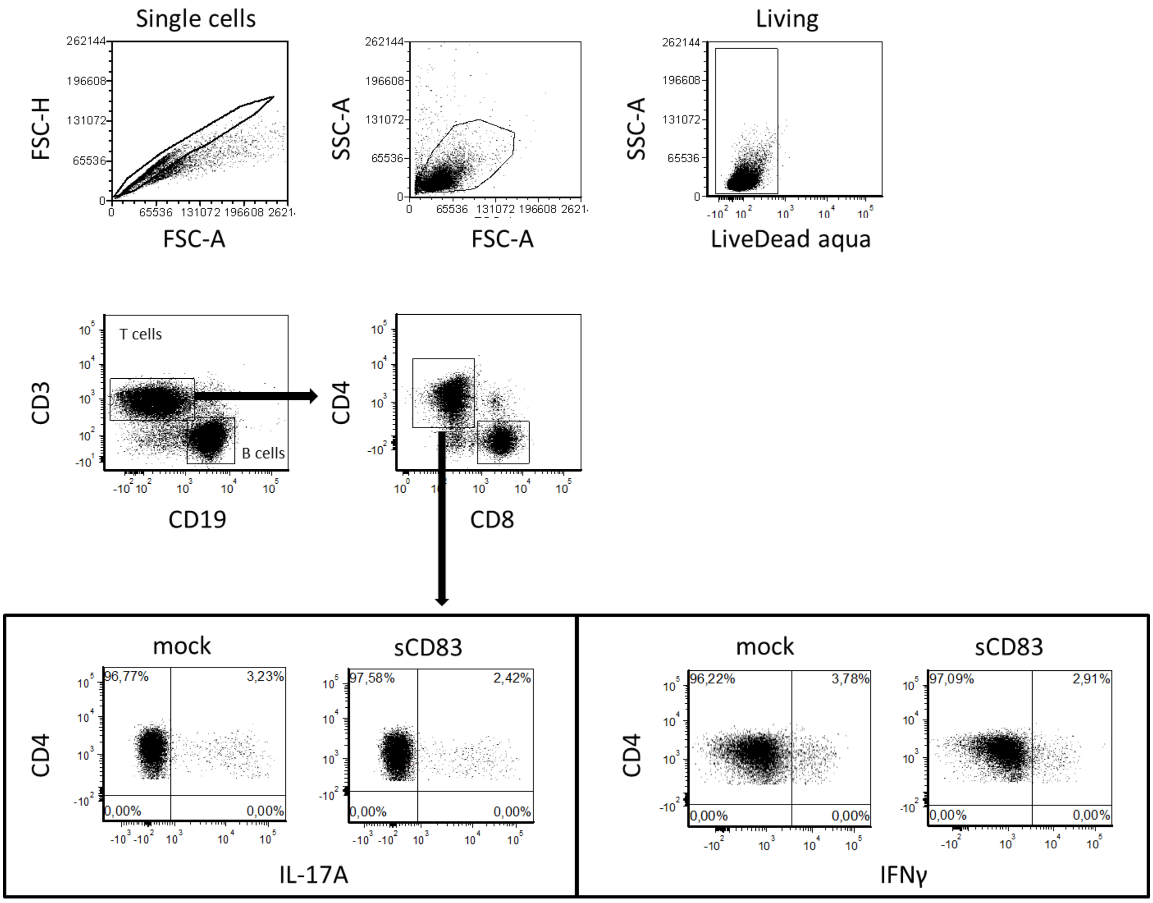

Suppl. Figure 3. Representative gating strategy used for flow cytometric analyses of Th-subsets. Both synovial and LN cells were isolated, stimulated with PMA/ionomycin for 6 h and analyzed regarding intracellular IL-17A and INFγ production in T cells by flow cytometry. Representative FACS plots for LN cells are shown.

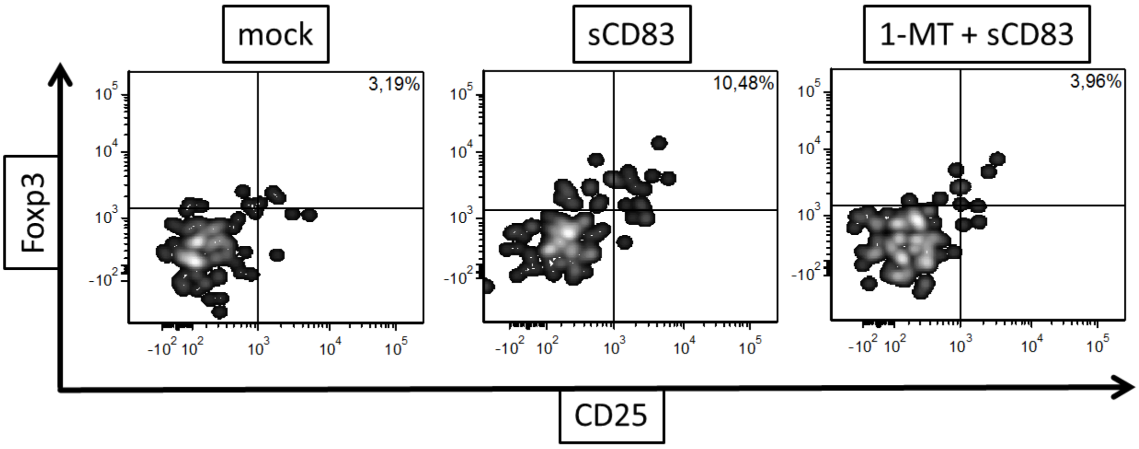

Suppl. Figure 4. Representative plots of flow cytometric analysis used for synovial Tregs. Synovial cells from AIA mice were isolated from the knee cavities and analyzed for their cellular composition by flow cytometry. Tregs were gated as Foxp3$^+$/CD25$^+$ cells within the CD4$^+$ population.

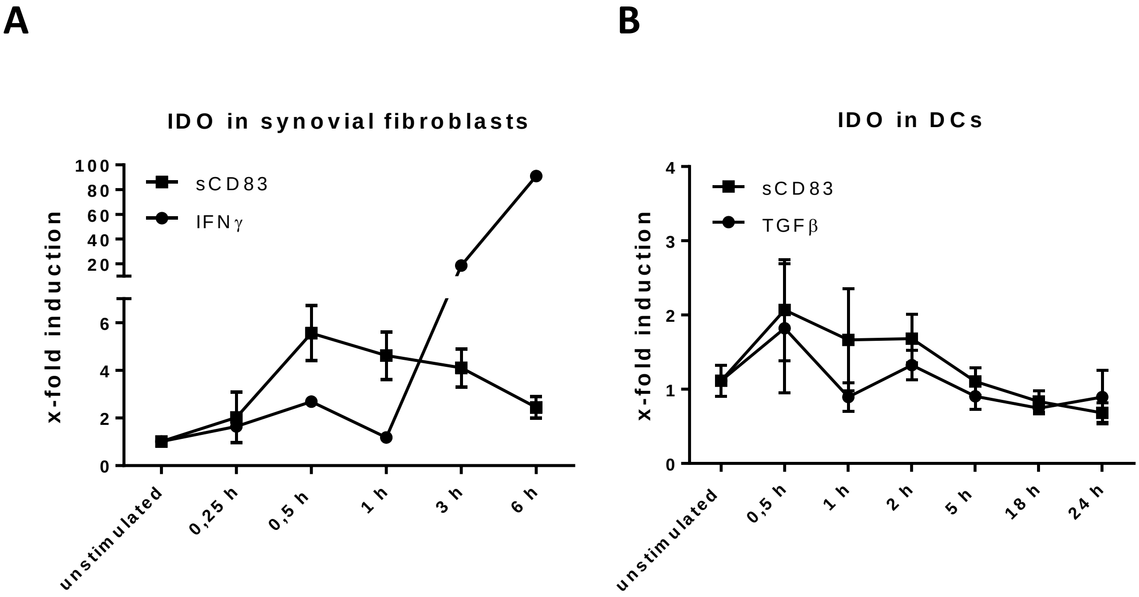

Suppl. Figure 5. sCD83 induced IDO expression in synovial fibroblasts and DCs. (A) Synovial fibroblasts from healthy C57BL/6 mice were cultivated in 24 well plates, treated with sCD83 (25 µg) at indicated time points and analyzed for their IDO expression by RT-PCR. (B) Analogues experiments were performed using DCs.
